# Development of Polymeric Nanoparticles for Blood–Brain Barrier Transfer—Strategies and Challenges

**DOI:** 10.1002/advs.202003937

**Published:** 2021-03-07

**Authors:** Weisen Zhang, Ami Mehta, Ziqiu Tong, Lars Esser, Nicolas H. Voelcker

**Affiliations:** ^1^ Drug Delivery, Disposition and Dynamics Monash Institute of Pharmaceutical Sciences Monash University 381 Royal Parade Parkville VIC 3052 Australia; ^2^ IITB Monash Research Academy Bombay Mumbai 400076 India; ^3^ Commonwealth Scientific and Industrial Research Organisation (CSIRO) Clayton VIC 3168 Australia; ^4^ Melbourne Centre for Nanofabrication Victorian Node of the Australian National Fabrication Facility Clayton VIC 3168 Australia; ^5^ Department of Materials Science and Engineering Monash University Clayton VIC 3800 Australia

**Keywords:** blood–brain barrier, drug delivery systems, microfluidic chips, neurological diseases, polymeric nanoparticles

## Abstract

Neurological disorders such as Alzheimer's disease, stroke, and brain cancers are difficult to treat with current drugs as their delivery efficacy to the brain is severely hampered by the presence of the blood–brain barrier (BBB). Drug delivery systems have been extensively explored in recent decades aiming to circumvent this barrier. In particular, polymeric nanoparticles have shown enormous potentials owing to their unique properties, such as high tunability, ease of synthesis, and control over drug release profile. However, careful analysis of their performance in effective drug transport across the BBB should be performed using clinically relevant testing models. In this review, polymeric nanoparticle systems for drug delivery to the central nervous system are discussed with an emphasis on the effects of particle size, shape, and surface modifications on BBB penetration. Moreover, the authors critically analyze the current in vitro and in vivo models used to evaluate BBB penetration efficacy, including the latest developments in the BBB‐on‐a‐chip models. Finally, the challenges and future perspectives for the development of polymeric nanoparticles to combat neurological disorders are discussed.

## Graphical abstract

This review highlights the development of polymeric nanoparticles for drug delivery to the central nervous system. The synthesis of promising polymeric nanoparticles is discussed with an emphasis on the effects of particle size, shape, and surface modification on blood–brain barrier (BBB) transfer. Moreover, current in vitro and in vivo models are discussed, including the latest advances in BBB‐on‐a‐chip models.

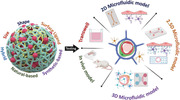

## 1 Introduction

### 1.1 Neurological Disorders

Neurological disorders that affect the brain and spinal cord are leading causes of morbidity and disability globally, with stroke being the second most common cause of death.^[^ ^1^ ^]^ Common neurological disorders include Alzheimer's disease, Parkinson's disease, Huntington's disease, motor neuron disease, multiple sclerosis, traumatic brain injury, stroke, and brain cancers. A comprehensive study of the global burden of diseases, injuries, and risk factors in 2016 estimated that 276 million people are suffering from neurological disability and about 9 million deaths occur from neurological disorders each year.^[^ ^1^ ^]^ With an aging and growing world population, there will be an even stronger demand for more effective management and treatment for neurological diseases.

Neurological disorders are predominantly associated with the central nervous system (CNS) that comprises the brain and spinal cord. Vital to the function and the regulation of the body, the CNS has three barriers: the cerebral microvascular endothelium (blood–brain barrier, BBB), the choroid plexus epithelium (blood–cerebrospinal fluid barrier), and the avascular arachnoid epithelium (cerebrospinal fluid–blood barrier). Due to these natural barriers, particularly the BBB, transporting pharmaceuticals into the CNS can be extremely difficult. Furthermore, neurons that harbor extensive cell–cell communication capabilities are key players in the CNS. Since neurons are extremely sensitive to temperature fluctuations, pathogens, and toxins, neurodegenerative diseases such as Parkinson's, Huntington's, and Alzheimer's disease that involve irreversable neuronal cell death are common.^[^ ^2^ ^]^ The irreversible process of neurodegeneration can also develop after ischemic or hypoxic conditions like stroke, birth asphyxia, and traumatic brain injuries resulting in slow and progressive loss of neuron functions. Furthermore, brain cancers, such as glioblastoma, are driven by oncogenic transformation of genetic and cellular factors in neurons and glial cells. In addition to primary tumors, secondary brain tumors involving brain metastases occur in 9–17% of adults with cancer.^[^ ^3^ ^]^

### 1.2 The Blood–Brain Barrier

**Figure 1:**
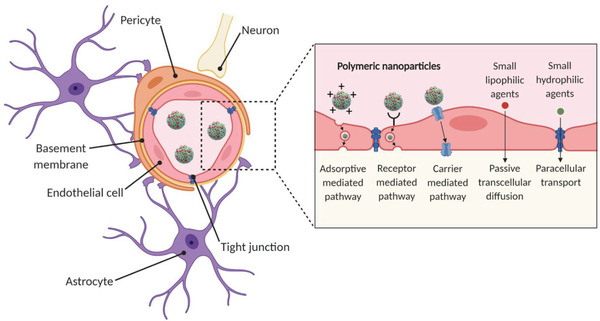
The neurovascular unit. Cross‐section schematic of a brain capillary depicting endothelial cells interconnected via tight junctions. On the brain side of the endothelial cells, the basement membrane surrounds the endothelial cells and embeds pericytes that span several endothelial cells. Astrocytic end‐feet are in contact with the endothelial cells. Neurons are present in the tissue of the brain. Polymeric nanoparticles are transported across the endothelium carrier via carrier‐mediated, receptor‐mediated, and adsorptive‐mediated pathways. Image is created with Biorender.

The BBB is a structural, functional, and physiological barrier that intricately regulates the movement of ions, nutrients, and cells between the blood and the brain. Anatomically, the BBB consists of cerebral endothelial cells, pericytes, astrocytes, and basement membrane (**Figure** **1**). The BBB acting together with neurons and glial cells forms the complete neurovascular unit (NVU) which is crucial for the function of the brain.^[^ ^4^ ^]^ The cerebral endothelial cells are non‐fenestrated, contain a large number of mitochondria, and form tight junctions that highly regulate the molecule transport across the endothelium. The inter‐endothelial space is characterized by the presence of transmembrane protein complexes composed of occludin, claudin, and junction adhesion molecules. These specialized tight junction proteins undertake homophilic interactions to form an intricate tight barrier that is exclusive to the cerebral endothelial cells. The apical side of the endothelial cell is exposed to the blood flow in the brain capillaries, while the basolateral side is exposed to the cerebrospinal fluid and is supported by the basement membrane (30–40 nm thick) composed of collagen type IV, laminin, heparin sulfate proteoglycans, fibronectin, and other extracellular matrix proteins.^[^ ^5^ ^]^

Aside from neurons, non‐neuronal cells (i.e., glial cells) also play an important role in the CNS. Pericytes are smooth muscle cells that span several endothelial cell lengths and form a discontinuous layer. They regulate the activity of endothelial cells and are likely to serve as macrophages during inflammation, thus providing a second line of defense after the tight junction of endothelial cells.^[^ ^6^ ^]^ On the other hand, astrocytes have a characteristic star‐shaped morphology and play a crucial role in enhancing the BBB integrity. The astrocytes secrete soluble factors, such as *β*‐2 microglobulin and transforming growth factor beta (TGF‐*β*), which upregulate the expression level of tight junction proteins on endothelial cells. An intact BBB restricts the entry of more than 98% of small molecule drugs and ≈100% of large molecule drugs.^[^ ^7^ ^]^ Under pathological conditions, such as neuroinflammation, traumatic brain injury, and brain cancers, the structural integrity and the function of the BBB can be compromised.^[^ ^8^ ^]^ Therefore, in brain cancers, the BBB is referred to as the blood–brain tumor barrier (BBTB), which is highly heterogeneous and characterized by numerous distinct features, including non‐uniform permeability and active efflux of molecules. However, in most low‐grade brain tumors and in the tumor periphery, the BBTB strongly resembles the BBB.^[^ ^9^ ^]^ Moreover, aging also contributes to dysfunctional barriers due to phenotypical changes of endothelial cells and decreased level of tight junction integrity.^[^ ^10^ ^]^ Finally, the BBB can be temporarily disrupted using techniques such as focused ultrasound in combination with microbubbles, focused radiation therapy, or chemical modifications using hypertonic solutions such as mannitol.^[^ ^11^ ^]^

### 1.3 Transport Mechanisms

Transport of substances through endothelial cells can be broadly divided into two categories: paracellular and transcellular pathways. The paracellular pathway involves the transport of molecules through the intracellular space between the cells. Small lipid‐soluble agents of low molecular weight (<400 Da), such as hormones, alcohol, and gases (CO_2_, O_2_) can passively diffuse through the plasma membrane of the endothelial cells.^[^ ^12^ ^]^ Although transport via the paracellular pathway is common in the peripheral capillaries, it is strictly limited in the BBB due to the presence of tight junctions which forces the majority of the transport through transcellular pathways.^[^ ^12^ ^]^ For example, nutrients and macromolecules are transported through the BBB via one of the three following transcellular pathways: carrier‐mediated transcytosis, receptor‐mediated transcytosis, or adsorptive‐mediated transcytosis.

#### 1.3.1 Carrier‐Mediated Transcytosis

Transporter protein carriers located on the luminal and basolateral sides of the endothelial cells are named nutrient and efflux transporter proteins, respectively. Nutrient transporter proteins are specific to solutes such as glucose, hormones, and amino acids. These solutes bind to their respective transporter proteins triggering a reversible conformational change. Upon cellular uptake of the solutes, they are transported to the basolateral side of the membrane, following high to low solute concentration gradient.^[^ ^13^ ^]^ For example, glucose transport is facilitated by the glucose transporter, GLUT1. On the other hand, a diverse range of ATP‐binding cassette transporters or efflux pumps are employed to actively transport non‐specific substrates and drugs out of the endothelial cells. These efflux pumps, which include P‐glycoprotein (P‐gp), multi‐drug resistance proteins (Mrp), and breast cancer resistance protein (Brcp), are found on the luminal side of the brain capillaries and bind to a variety of substrates, and they effectively prevent drug accumulation in endothelial cells and hamper the transport of drugs to the brain.^[^ ^14^ ^]^

#### 1.3.2 Receptor‐Mediated Transcytosis

Cerebral endothelial cells express highly specialized receptors for macromolecules such as hormones, enzymes, and plasma proteins. The three most‐studied ligands important for BBB transport are insulin, transferrin, and low‐density lipoprotein (LDL)‐cholesterol, which bind to insulin, transferrin (Tf), and LDL receptors on endothelial cells, respectively.^[^ ^15^ ^]^ On the luminal side of the endothelium, ligands bind to the receptors on the plasma membrane and are internalized through the formation of vesicles. These vesicles are subsequently transported through the cytoplasm of the cells and then release the ligands on the basolateral side. The internalization of cargos through endocytosis can occur via clathrin‐ or caveolin‐dependent pathways. Transcytosis of most ligands, such as LDL‐1, transferrin, and insulin follows the clathrin‐dependent pathway.^[^ ^12^ ^]^ This is a five‐step process that involves nucleation (binding of the cargo to the plasma membrane), cargo selection (initiation of clathrin‐coated pit formation), the assembly of the clathrin coat, membrane scission, and disassembly of the clathrin coat. Only a few compounds, such as folate, undertake the caveolin‐dependent pathway that is mediated by the caveolin protein and results in the formation of uncoated vesicles. However, this pathway is more relevant to leaky BBB in neurological diseases.^[^ ^12^ ^]^

#### 1.3.3 Adsorptive‐Mediated Transcytosis

Drugs or substrates that are positively charged can undertake adsorptive‐mediated transcytosis. It is triggered by electrostatic interactions between the positively charged substrate surface, usually polycationic proteins (e.g., protamine) and the negatively charged heparin sulfate proteoglycans present on the plasma membrane surface of the endothelial cells.^[^ ^16^ ^]^ This is a relatively slower process in comparison to carrier‐ or receptor‐mediated transport and has a lower transport capacity.

### 1.4 Strategies for BBB Transfer

Currently, the development of drugs for the treatment of neurological diseases is limited by the complex challenges that are posed by the neurovascular unit. One of the main obstacles is the presence of the BBB, which makes it difficult to transport the drugs to the brain tissue. The ability of drugs to penetrate the BBB depends on the drug size, hydrophilicity, lipid solubility, transport pathway, and degree of dissociation of the drug molecules.^[^ ^17^ ^]^ Conventional approaches that use the drug molecules in their free form have demonstrated poor BBB penetration due to the presence of efflux pumps on the endothelial cells that strictly regulate the movement of drug molecules. This results in ineffective delivery of the drug molecules to the target cells in the brain.

**Figure 2:**
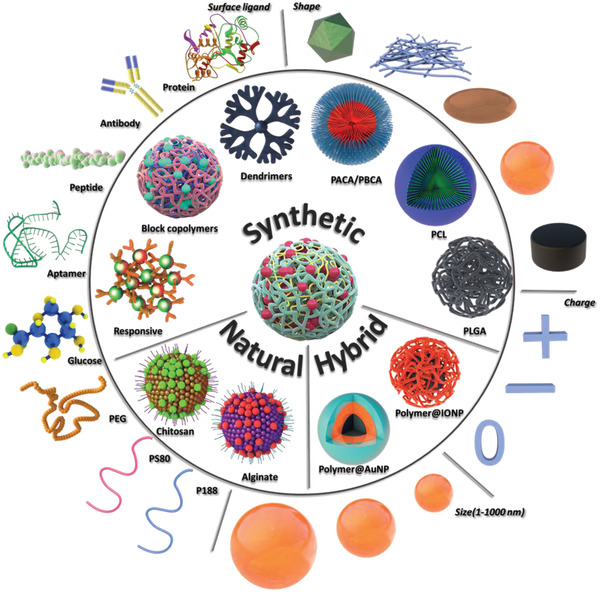
An overview of polymeric nanoparticles developed for BBB penetration and their tunable parameters: surface (charge), size, and shape. PACA: poly(alkyl cyanoacrylate); PBCA: poly(butyl cyanoacrylate; PCL: poly‐*ε*‐caprolactone; PLGA: poly(lactic‐*co*‐glycolic acid); Polymer@AuNP: polymer‐coated gold nanoparticle; Polymer@IONP: polymer‐coated iron oxide nanoparticle; PEG: polyethylene glycol; PS80: polysorbate 80; and P188: poloxamer 188. Image is created with Biorender.

Nanotechnology has immense potential in addressing the complex needs for the treatment of neurological disorders, such as the penetration of the BBB and consequential drug delivery to cells of interest.^[^ ^18^ ^]^ This can be achieved using nanosized drug delivery systems that can be specifically engineered (e.g., composition, size, shape, and surface ligands) to shuttle drugs across the BBB. For example, rational designs of nanoparticles can enhance circulation time in the brain capillaries and can take advantage of transcytosis pathways using different surface strategies (Trojan horse strategy). Furthermore, nanoparticles have the ability to escape the P‐gp efflux pumps owing to the presence of specific ligands engineered onto the particle surface.^[^ ^19^ ^]^ Numerous drug delivery systems have been developed to improve drug delivery and are usually categorized as either organic or inorganic nanoparticles. The most common organic nanoparticles are the liposomes, polymeric nanoparticles, and lipid nanoparticles, while examples of inorganic nanoparticles are the iron oxide nanoparticles, gold particles, and quantum dots.^[^ ^17^ ^]^ Polymeric nanoparticles, in particular, are a promising choice as drug delivery platform for CNS targeting, due to their tunable architecture (10 to 1000 nm), non‐toxicity, biocompatibility, and controllable drug release.^[^ ^20^ ^]^ These polymeric nanoparticles can be easily modified with specific ligands that target the receptors on the endothelial cells, resulting in improved transcytosis efficiency (**Figure** **2**).^[^ ^18^ ^]^ Furthermore, polymeric nanoparticles have an increased circulation time and can be biodegradable.^[^ ^21^ ^]^ After cellular uptake and internalization, the polymeric matrix can be triggered to release the drug, resulting in a protected, prolonged, and targeted therapeutic effect. Polymeric nanoparticles are versatile to be able to deliver a wide range of drugs, for example, via hydrophilic, hydrophobic, or electrostatic interactions, and via responsive covalent bonds.^[^ ^22^ ^]^

### 1.5 Testing Models

The first technique to isolate rat brain endothelial cells and culture them in vitro was pioneered by Ferenc Joό in 1996.^[^ ^23^ ^]^ Since then, there has been substantial development in the understanding of the physio/pathological conditions of the brain and the mechanism of molecule transport to the brain. Choosing the right research model for drug (and drug complex) development is crucial because it provides valuable insights into translational research. Various in vitro, in vivo, and in silico models have been developed to study the transport of drugs and drug complexes across the BBB (**Table** **1**). In vivo models offer physio/pathological conditions that can be evaluated for pharmacokinetics and pharmacodynamics (PK/PD) studies. However, they are costly and time consuming and do not translate directly into human conditions, due to species‐to‐species differences.^[^ ^24^ ^]^ For example, differences in the capacity of plasma protein binding to the drug/substrate result in different barrier tightness. On the other hand, in vitro models are easy to set up, offer high throughput studies at a low cost, and are capable of real‐time microscopic measurements. Various BBB microfluidic models have been established to mimic the physiological conditions of the brain and to investigate therapeutic targets for penetrating the BBB to combat neurological diseases.^[^ ^25^ ^]^ Although the majority of the in vitro BBB models are focused on the “non‐pathological” brain condition, in which the integrity of the BBB is maintained, some BBB models have been explored to simulate the “pathological” brain conditions with compromised BBB integrity.^[^ ^26^ ^]^ In silico models reduce the cost of setting up in vitro or in vivo experiments by simulating the drug compound efficacy and predict its permeability across the BBB.^[^ ^27^ ^]^

**Table 1:** Advantages and limitations of the various BBB models

BBB models/features	2D static models	Dynamic in vitro model	Microfluidic models	In vivo models
Ease of setup	Easy	Moderate	Moderate	Extensive skills required
Cost effectiveness	Minimal	Reasonable	Reasonable	Expensive
Co‐culture	Possible up to tri‐culture	Possible up to bi‐culture	Possible to setup entire neurovascular unit	Native
Geometry	2D, flat morphology	3D, cylindrical	3D, cylindrical	Native
Permeability/TEER measurements^a)^	Low TEER values	Moderate TEER values	High TEER values	Invasive, difficult to measure
Imaging capability	Limited	Yes	Yes	Challenging, special instrument and skills required
3D organization	No	Limited	Yes	Native
Mechanical stimulus	No	Yes, (shear stress induced by pulsatile flow)	Yes, (shear stress induced by interstitial and pulsatile flow)	Yes, biological
High‐throughput drug screening	Yes	No	Moderate	Highly expensive due to large number of animals required
Personalized medicine	Possible	No	Yes	No
Real‐time measurement	Limited	Limited	Yes	Yes
PK/PD profiling^b)^	No	No	No	Yes

^a)^ TEER, trans‐endothelial electrical resistance

^b)^ PK/PD, pharmacokinetics/pharmacodynamics

In this review, we first discuss the polymeric nanoparticle systems that have been used for drug delivery to the CNS, including their synthesis methods and the latest advances. Next, we describe how their properties (i.e., surface, size, and shape) can be tuned to increase BBB penetration. Moreover, we critically review the in vitro and in vivo models used to evaluate BBB penetration efficacy of polymeric nanoparticles, including the latest developments in BBB‐on‐a‐chip models. Finally, we give an overview of the challenges and future perspectives for evaluating polymeric nanoparticles in clinically relevant BBB testing models.

## 2 Polymeric Nanoparticle Systems for BBB Transfer

Polymeric nanoparticles can be prepared from a plethora of monomers and using various polymerization techniques, and their properties can be tuned depending on their specific applications. In this section, we first discuss the most common polymeric nanoparticle systems that have been exploited for brain targeting, namely synthetic polymeric nanoparticles, natural‐based polymeric nanoparticles, and hybrid nanoparticles. We describe the synthesis methods of each polymer and its respective nanoparticle, drug loading, surface functionalization, and their suitability for drug delivery crossing the BBB. Thereafter, the effects of different nanoparticle parameters on BBB crossing are discussed: particle surface ligands, charge, size, and shape.

### 2.1 Synthetic Polymeric Nanoparticles

#### 2.1.1 Poly(Alkyl Cyanoacrylate)

**Figure 3:**
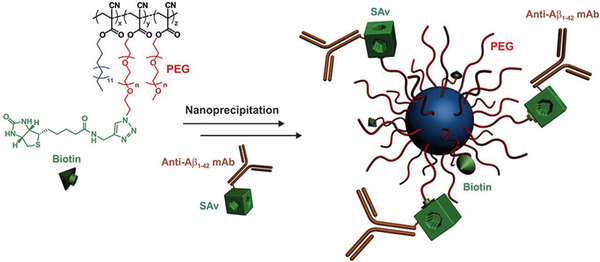
Schematic overview of the synthesis of PACA nanoparticles for BBB crossing which were prepared by the nanoprecipitation of PEGylated PACA polymers functionalized with biotin. Thereafter, the nanoparticles were functionalized with monoclonal anti‐A*β*1–42 antibody. Reproduced with permission.^[^ ^38^ ^]^ Copyright 2018, Elsevier.

Poly(alkyl cyanoacrylate)s (PACA) are commonly known as superglues and have been commonly used as suture materials.^[^ ^28^ ^]^ PACA nanoparticles were first developed by Couvreur et al. in 1972.^[^ ^29^ ^]^ They have low toxicity^[^ ^30^ ^]^ and are degraded by esterases from the pancreatic juice in the intestinal tract (oral administration) or by serum esterases in the blood.^[^ ^31^ ^]^ The degradation time is in the order of hours and can be controlled by modifying the alkyl side chain length. For example, polymers with a longer side chain (e.g., octyl) degrade slower than shorter (e.g., butyl) side chain (PBCA).^[^ ^32^ ^]^ Moreover, the choice of side chain also affects the toxicity profile.^[^ ^33^ ^]^ PACAs can be synthesized via several polymerization techniques such as free radical, anionic, and zwitterionic polymerization,^[^ ^34^ ^]^ and PACA nanoparticles are prepared either by polymerization in aqueous acidic phase or via interfacial emulsion polymerization.^[^ ^28^ ^]^ PACA nanoparticles can be functionalized by esterification of cyanoacetic acid with, for example, polyethylene glycol (PEG)‐amine, folic acid, or drugs to create cyanoacetate esters that can then be polymerized. A variety of drugs have been loaded by encapsulation or adsorption including hydrophilic or poorly soluble molecules, peptides, proteins, and nucleic acids.^[^ ^34^, ^35^ ^]^ For brain delivery, PACA nanoparticles have been modified with PEG to escape macrophage uptake or with polysorbate 80 to improve their ability to penetrate the BBB.^[^ ^36^ ^]^ In another study, PACA nanoparticles were decorated with anti‐A*β*1–42 antibody^[^ ^37^ ^]^ and a significant increase in A*β* level was detected in the plasma, leading to memory recovery in an Alzheimer's disease mouse model (**Figure** **3**).^[^ ^38^ ^]^ These surface modifications will be discussed in more detail in Section 3.1. In fact, several PACA formulated nanoparticles have been investigated in clinical trials, although not yet for CNS diseases. For instance, PACA nanoparticles loaded with doxorubicin or mitoxantrone have been tested in patients with refractory solid tumors or hepatocellular carcinoma, respectively.^[^ ^31^, ^39^ ^]^ A phase II trial was discontinued due to severe acute respiratory distress events;^[^ ^40^ ^]^ however, this was solved by changing the administration modality from the intra‐hepatic arterial route to a slow infusion by the intravenous route. Unfortunately, a phase III trial did not show any added survival benefit for patients compared to the best standard of care.^[^ ^31^ ^]^ One of the reasons for the lack of clinical translation has been postulated by the variability in drug entrapment rate and release profiles.^[^ ^39^, ^41^ ^]^

#### 2.1.2 Poly(Lactic‐*co*‐Glycolic Acid)

Poly(lactic‐*co*‐glycolic acid) (PLGA) is a family of linear copolymers that can be prepared with different ratios of its constituent monomers, glycolic acid and lactic acid.^[^ ^42^ ^]^ PLGA has been approved by the United States Food and Drug Administration (FDA) for medical uses such as sutures, drug delivery systems, and biomaterials (e.g., screws^[^ ^42^ ^]^). The PLGA copolymers are non‐toxic and biodegradable via hydrolytic de‐esterification followed by the clearance of their monomeric anions, glycolate and lactate.^[^ ^43^ ^]^ The degradation rate, mechanical strength, degree of crystallinity, and thus drug loading and release kinetics can be precisely controlled by changing the lactic acid to glycolic acid ratio. Whereas poly(lactic acid) (PLA) is a crystalline hydrophobic polymer due to its methyl sidechains, poly(glycolic acid) (PGA) is a stiff and hydrophilic polymer with a low mechanical strength.^[^ ^44^ ^]^ Consequently, PLGA copolymers with a higher PLA:PGA ratio are more hydrophobic and thus have a lower degradation and drug release rate. For example, the biodegradation rate of a 50:50 ratio is around 1 week (also dependent on molecular weight) as compared to a degradation rate up to 18 weeks for pure PLA.^[^ ^45^ ^]^

**Figure 4:**
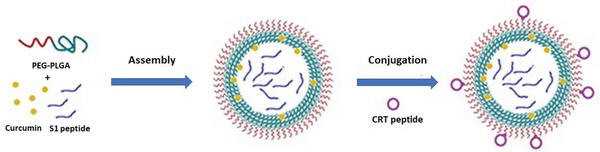
Preparation of PLGA nanoparticles. The PLGA nanoparticles were loaded with curcumin, S1 peptide (an A*β* generation inhibitor) and brain targeting peptide, CRT. Reproduced with permission.^[^ ^54^ ^]^ Copyright 2017, Impact Journals.

PLGA can be synthesized using several techniques: the polycondensation process,^[^ ^45^ ^]^ ring opening polymerization,^[^ ^46^ ^]^ and Segmer assembly polymerization.^[^ ^47^ ^]^ PLGA nanoparticles can be obtained using methods such as emulsion, nanoprecipitation, solvent co‐evaporation, and spray‐drying from PLGA copolymer.^[^ ^48^ ^]^ Non‐spherical nanoparticles (e.g., cylindrical shape) can also be prepared using soft lithography methods.^[^ ^49^ ^]^ Surface modifications can be introduced via the terminal carboxylic acid groups, for example, creating diblock (PEG‐*b*‐PLGA) or triblock copolymers, PLGA‐*b*‐PEG‐*b*‐PLGA,^[^ ^50^ ^]^ or introducing targeting moieties such as folic acid or antibodies.^[^ ^51^ ^]^ Therefore, a wide range of drug molecules have been incorporated in PLGA nanoparticles including chemotherapeutics, antibiotics, anti‐inflammatory drugs, and proteins.^[^ ^48^ ^]^ Numerous PLGA formulations have been studied for crossing the BBB.^[^ ^52^ ^]^ For example, PLGA nanoparticles decorated with a cyclic transferrin‐targeting peptide and loaded with A*β* generation inhibitor peptide and curcumin showed improved spatial memory and recognition in transgenic mice (**Figure** **4**).^[^ ^53^ ^]^ Moreover, two non‐CNS targeting PLGA‐formulations have been clinically approved. Genexol‐PM was approved for the treatment of head and neck cancer and breast cancer in South Korea in 2006, while Nanoxel was approved for various cancers in India in 2007.^[^ ^54^ ^]^ In addition, phase II clinical trials were successfully carried out using PGLA nanoparticles loaded with docetaxel (BIND‐014) and targeting a prostate specific membrane antigen in prostate cancer in 2016.^[^ ^54^ ^]^

#### 2.1.3 Poly‐*ε*‐Caprolactone

Poly‐*ε*‐caprolactone (PCL) is a biodegradable, FDA‐approved polyester^[^ ^55^ ^]^ and has been used among several applications: sutures (Monocryl), implants (e.g., 3D printed OsteoPlug for covering burr holes), contraceptive devices, and as drug delivery systems.^[^ ^56^ ^]^ Chemically, PCL is composed of repeating units of hexanoate and can be degraded in the body by hydrolysis into 6‐hydroxycaproic acid,^[^ ^57^ ^]^ which can be further transformed into adipate^[^ ^58^ ^]^ and then catalyzed to CO_2_.^[^ ^59^ ^]^ PCL is synthesized either by ring‐opening polymerization of *ε*‐caprolactone or via condensation polymerization of 6‐hydroxyhexanoic acid. Block copolymers containing PCL have been widely used, for example, PCL‐*b*‐PLGA (by grafting onto terminal di‐hydroxyl groups of PCL)^[^ ^47^ ^]^ or PEG‐*b*‐PCL (by ring opening polymerization of *ε*‐caprolactone with methoxy‐PEG as initiator).^[^ ^60^ ^]^ PCL‐based nanoparticles are predominantly synthesized using diblock PEG‐*b*‐PCL copolymers due to the insolubility of PCL in water. These nanoparticles can be prepared via standard methods such as solvent‐displacement, film dehydration, emulsion, and microfluidics.^[^ ^61^ ^]^ PCL‐based nanoparticles have also been investigated for drug delivery for neurological diseases.^[^ ^62^ ^]^ For instance, peptide‐functionalized PEG‐PCL micelles displayed significantly increased transport ratios in an in vitro BBB model and an enhanced accumulation in an intracranial glioma tumor‐bearing in vivo model.^[^ ^63^ ^]^ However, the main limitation of PCL to be used as a drug delivery system is its low degradation rate (up to 1 year).^[^ ^64^ ^]^ This drawback could potentially be overcome by modifying the molar mass or coating with other polymers such as copolymer with PLA.^[^ ^65^ ^]^ Until now, no PCL‐based nanoparticles have been clinically approved.^[^ ^66^ ^]^

#### 2.1.4 Polyamidoamine Dendrimers

Dendrimers are biocompatible 3D polymeric macromolecules that consist of tree‐like branches extending from a central core and have a corona with reactive functional groups.^[^ ^67^ ^]^ Their size is measured in generations, based on the layer‐by‐layer structure by which they are synthesized.^[^ ^68^ ^]^ Different types of dendrimers have been developed and the most prominent one is based on polyamidoamine (PAMAM). PAMAM can be synthesized using either a divergent or convergent method using Michael addition reactions followed by amidations. Other than amines, other surface functional groups such hydroxyl (−OH)^[^ ^69^ ^]^ or carboxylic acid (−COOH)^[^ ^70^ ^]^ can also be incorporated. These functional groups can render PAMAM dendrimers more water soluble, limit opsonization, and reduce clearance by the mononuclear phagocyte system (MPS).^[^ ^71^ ^]^ Drugs are loaded to PAMAM via physical entrapment in the hydrophobic cavities or via conjugation to the surface functional groups.^[^ ^72^ ^]^ As PAMAM dendrimers are usually smaller than 15 nm (depending on generation), they have been explored as another candidate drug delivery system for brain delivery.^[^ ^73^ ^]^ For instance, dendrimers were shown to be able to cross the compromised BBTB of rodents with malignant glioma,^[^ ^74^ ^]^ neuroinflammatory disease such as cerebral palsy,^[^ ^75^ ^]^ and traumatic brain injury.^[^ ^76^ ^]^ Moreover, PAMAM dendrimers (generation three) coated with a streptavidin adapter were shown to pass through the intact BBB via transcytosis, and slightly protonated G4 PAMAM dendrimers (10% amine) were able to reach the brain in healthy mice.^[^ ^77^ ^]^ Despite the high medical expectations and research effort, clinical translation for dendrimers has been limited with only Starpharma's polylysine dendrimer‐based antimicrobial treatment approved for healthcare products.^[^ ^67^ ^]^ Nevertheless, efforts have been made to significantly shorten dendrimer synthesis pathways and optimize the particle design, for example, amending multiple functional groups and incorporation of inner core functionalization to enable a higher drug loading.

#### 2.1.5 Novel Synthetic Polymers

The aforementioned polymer‐based drug delivery systems have been investigated for several decades, however, with very limited clinical success. Therefore, recent research has been directed to other polymer systems to further enhance nanoparticle properties, such as drug loading, targeting, and controlled release kinetics. For example, pH‐responsive polymersomes based on the block copolymer poly(ethylene glycol) ethyl methacrylate‐block‐poly(2‐(diisopropylamino)ethyl methacrylate) (POEGMA‐*b*‐PDPA) have been shown to improve antibody delivery to the brain.^[^ ^78^ ^]^ The polymersomes were conjugated with a peptide targeting the LDL‐related protein (LRP‐1). Interestingly, the study showed that LRP‐1 mediated transcytosis did not involve endocytic sorting and consequently pH‐driven degradation, and enabled intact nanoparticle transportation to CNS cells where the protein was released by endocytic acidification.^[^ ^78^ ^]^ Drug loading efficiency can also be improved using other polymers. For example, transferrin‐coated nanoparticles consisting of the diblock copolymer PLA‐d‐alpha‐tocopheryl PEG 1000 succinate had a higher drug loading than PLGA nanoparticles,^[^ ^79^ ^]^ and many other block copolymers have also been investigated. For example, a library of polymers based on (P(N‐(2‐hydroxypropyl)‐methacrylamide) (PHPMA) and P(laurylmethacrylate) (PLMA) were synthesized and were investigated for their BBB crossing capability. It was shown that a random copolymer containing 10% LMA was the most promising system due to the anchoring of the fatty acid‐like chains on the membrane.^[^ ^80^ ^]^ Moreover, a dual targeting 2‐deoxy‐d‐glucose functionalized PEG‐*co*‐poly(trimethylene carbonate) also exhibited enhanced BBB crossing.^[^ ^81^ ^]^

**Figure 5:**
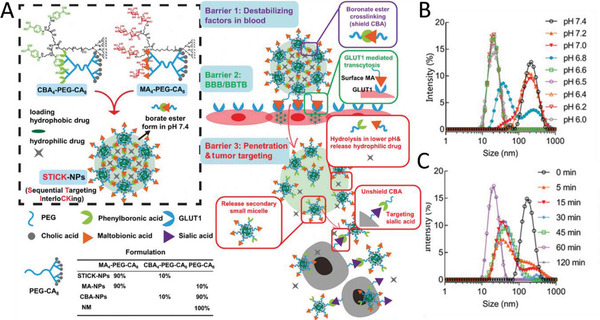
Sequential Targeting Interlocking (STICK) nanoparticles for BBB and blood–brain tumor barrier (BBTB) crossing. A) Synthesis scheme of STICK nanoparticles that consist of cross‐linked telodendrimer micelles functionalized with maltobionic acid as BBB targeting ligand. B) The size of the nanoparticles is pH‐dependent, and pH 6.8 appears to be the cut‐off value for triggering micellular transformation. C) Moreover, the size change is also time dependent (pH 6.5). Reproduced with permission.^[^ ^82^ ^]^ Copyright 2020, John Wiley & Sons.

An interesting concept using a polymeric nanoparticle for sequential targeting based on cross‐linked telodendrimer micelles has been developed (**Figure** **5**).^[^ ^82^ ^]^ Maltobionic acid (a glucose derivative) was conjugated on the nanoparticle surface to promote GLUT1 receptor mediated BBB transcytosis. Upon exposure to an acidic extracellular pH (e.g., in solid tumors), the intrinsic boronate ester cross‐linkages are cleaved, transforming the nanoparticle into smaller secondary nanoparticles (Figure 5B,C) with newly unshielded surface carboxyphenylboronic that promoted tumor cell uptake.

Positively charged polymeric nanoparticles have also been developed to enhance delivery of negatively charged gene therapeutics such as DNA and small‐interfering RNA (siRNA).^[^ ^83^ ^]^ For example, poly(ethylene imine) (PEI)‐based nanoparticles were prepared and modified with glutathione to promote BBB penetration. The block copolymers, containing 80% primary or secondary amine groups, respectively, were investigated for penetration using a microfluidically perfused biochip, showing that secondary amines enhanced better BBB crossing performance.^[^ ^84^ ^]^

**Figure 6:**
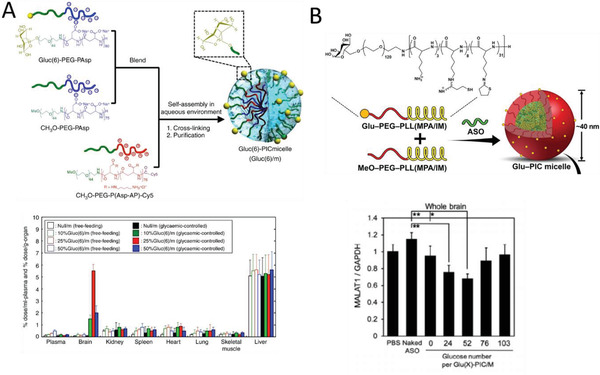
A) Synthesis of polyion complex (PIC) micelles with different ratios of glucose on the surface (Gluc(6)/m) and their biodistribution in mice under different feeding conditions 48 h after injection. Open and closed bars show free‐feeding and glycemic‐controlled groups, respectively. Reproduced with permission.^[^ ^85^ ^]^ Copyright 2017, Nature Publishing Group. B) PIC micelles for the delivery of antisense oligonucleotides (ASO) and the effect of glucose numbers on knock‐down. Reproduced with permission.^[^ ^87^ ^]^ Copyright, 2020, Wiley‐VCH.

The Kataoka group explored polymeric micelles for BBB penetration (**Figure** **6**).^[^ ^85^, ^86^ ^]^ These polymeric micelles were formulated using opposite charged pairs of PEG‐based block ionomers, that is, negatively charged PEG–poly(*α*,*β*‐aspartic acid) (PEG–PAsp) and positively charged PEG–poly([5‐aminopentyl]‐*α*,*β*‐aspartamide) (PEG–P(Asp‐AP)).^[^ ^85^ ^]^ These ionomers were blended with glucose‐modified PEG‐PAsp to investigate the effect of the number of targeting moieties on BBB transfer efficiency (Figure 6A). Delivery of antisense oligonucleotides using a bespoke polymer system was further investigated.^[^ ^87^ ^]^ A polyion complex (PIC) micelle self‐assembled from PEG*‐b*‐poly(l‐lysine) modified with 3‐mercaptopropyl amidine and 2‐thiolaneimine block copolymer was synthesized and the antisense oligonucleotides were immobilized through electrostatic interactions in the polymer core (Figure 6B). Disulfide crosslinking was introduced in the micelle core by partially derivatizing the side chain of the poly(l‐lysine) segment with sulfhydryl groups to improve its stability in the blood while stimulating release in the reductive condition in the brain. Finally, LDL receptor family‐targeted polymersomes were recently synthesized by blending PEG‐*b*‐poly(trimethylene carbonate‐*co*‐dithiolane trimethylene carbonate)‐*b*‐polyethylenimine (PEG‐P(TMC‐DTC)‐PEI) and apolipoprotein E peptide conjugated PEG‐P(TMC‐DTC). They demonstrated that these saporine‐loaded targeted polymersomes could cross the BBB in an in vitro BBB model and that systematic administration resulted in a complete growth inhibition in an orthotopic glioblastoma model.^[^ ^88^ ^]^

### 2.2 Natural Polymeric Nanoparticles

The use of synthetic polymers can sometimes be restricted due to their cost, purity, and undesirable toxicity profiles.^[^ ^89^ ^]^ Therefore, nanoparticles based on naturally occurring polymers have also been explored as an alternative approach in brain drug delivery owing to their low toxicity, sustainability, low cost, and unique physicochemical characteristics including biodegradability.^[^ ^90^ ^]^

#### 2.2.1 Chitosan

**Figure 7:**
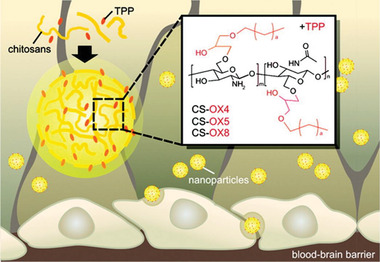
Chitosan‐based nanoparticle for BBB crossing. O‐substituted alkyl‐glyceryl chitosan nanoparticles were prepared with systematically varied alkyl chain lengths (butyl‐OX4, pentyl‐OX5, octyl‐OX8) and sodium tripolyphosphate (TPP) to enhance BBB penetration. Reproduced with permission.^[^ ^98^ ^]^ Copyright 2012, American Chemical Society.

Chitosan is a cationic linear polysaccharide and is one of the most commonly used natural polymer‐based nanoparticles for drug delivery due to its low cost, biodegradability, and availability in a wide range of molecular weights.^[^ ^91^ ^]^ It also has unique inherent biological properties, such as anti‐cancer, antimicrobial, and antioxidant characteristics.^[^ ^92^ ^]^ Chitosan consists of randomly distributed *β*‐(1,4)‐linked d‐glucosamine and *N*‐acetyl‐d‐glucosamine units and is prepared by partial *N*‐deacetylation of chitin, a natural polymer extracted from crustaceans or fungi.^[^ ^93^ ^]^ Chitosan has three types of functional groups (i.e., amine, primary and secondary hydroxyl) which can be exploited for various chemical modifications (**Figure** **7**). Its biodegradability can be tuned by varying molecular weight, degree of deacetylation, and chemical modifications.^[^ ^92^ ^]^ Chitosan nanoparticles can be prepared using a variety of methods including chemical cross‐linking, ionic gelation and microfluidic synthesis.^[^ ^94^ ^]^ These natural nanoparticles have shown promise in brain delivery due to their positive charge, which enhanced cell uptake and suitable for loading with negatively charged therapeutics.^[^ ^95^ ^]^ For example, antibody‐modified PEG‐chitosan nanoparticles showed a high brain uptake that was attributed to the synergy of the antibody and positive chitosan charge.^[^ ^96^ ^]^ Nevertheless, chitosan nanoparticles have limitations such as a low drug loading efficiency of hydrophobic substrates^[^ ^97^ ^]^ and poor control over molecular weight. In fact, drug loading efficiency has been shown to be improved using chemical modifications such as grafting palmitic acid.^[^ ^90b^ ^]^

#### 2.2.2 Alginate

Alginate is an anionic linear unbranched polysaccharide and is extracted from brown seaweed (phaeophyceae). It is a random copolymer consisting of *β*‐d‐mannuronic acid and *α*‐l‐guluronic acid via 1,4‐glycosidic linkages.^[^ ^99^ ^]^ Alginate is a non‐immunogenic substance that has been approved by the FDA^[^ ^100^ ^]^ and has been used for wound healing, drug delivery, and tissue engineering applications.^[^ ^101^ ^]^ Alginate has hydroxyl and carboxylic acid functional groups, which are exploited to introduce highly reactive functional groups (e.g., aldehyde groups) or introduce chemical (e.g., phosphate or sulfate) or biochemical (e.g., amino acids) groups that can increase its biointegration and bioaffinity properties.^[^ ^102^ ^]^ Alginate nanocapsules and nano‐aggregates are prepared by complexation (using cationic compounds or divalent cations like Ca^2+^).^[^ ^90b^ ^]^ Alginate nanospheres are made using a water/oil emulsion method coupled with gelation.^[^ ^103^ ^]^ Responsive alginate nanoparticles (pH or redox) can be synthesized by mixing with other polymers such as poly[(2‐dimethylamino) ethyl methacrylate]^[^ ^104^ ^]^ or employing disulfide cross‐links.^[^ ^90^, ^105^ ^]^ Brain delivery using alginate formulated nanoparticles have recently been reported. For example, alginate‐cholesterol micelles coated with lactoferrin were shown to be able to deliver a neuroprotective steroid to the brain,^[^ ^106^ ^]^ and alginate nanoparticles cross‐linked with chitosan were shown to improve brain delivery of an anti‐depressant.^[^ ^107^ ^]^ Furthermore, doxorubicin–alginate nanocomplexes with chitosan matrices showed enhanced uptake into the brain of rabbits.^[^ ^108^ ^]^

### 2.3 Hybrid Nanoparticles

Polymers have also been shown as a vital coating material for inorganic or lipid nanoparticles to form hybrid nanoparticles for brain delivery. For example, PEGylated liposomes (e.g., DaunoXome and Onivyde) and dextran coated Feridex iron oxide nanoparticles are FDA‐approved nanomedicines.^[^ ^66^ ^]^ Foremost, PEG is used to improve blood circulation time and colloidal stability, including in CNS delivery. For example, an interesting system to improve magnetic resonance imaging (MRI) delineation of the periphery of brain tumors was recently published. The periphery of brain tumors has an intact BBB and therefore receptor‐mediated transcytosis is required to pass the BBB.^[^ ^109^ ^]^ Gold nanoparticles were coated with PEG via a pH‐sensitive hydrazone bond and decorated with gadolinium‐chelates (click functional groups) and an LRP‐1 recognizing peptide. Upon BBB penetration, the hybrid nanoparticles aggregated in the acidic tumor environment after PEG cleavage, which resulted in increased MRI signals. PEGylated liposomes were also exploited to co‐deliver temozolomide and bromodomain inhibitor therapy, which showed a reduction in tumor burden and protection from the effects of systemic drug toxicity.^[^ ^110^ ^]^ PEI is also used in hybrid nanoparticles to enhance drug loading, for example, for siRNA (gold nanoparticles)^[^ ^111^ ^]^ and doxorubicin (iron oxide nanoparticles).^[^ ^112^ ^]^ Natural polymers have been used in hybrid nanoparticles as well. Next to the ubiquitous dextran to coat, for example, iron oxide nanoparticles,^[^ ^113^ ^]^ also other natural polymers such as chitosan have been exploited or combinations of above.^[^ ^97^, ^114^ ^]^ For example, hybrid nanoparticles consisting of an iron oxide core and an outer shell of chitosan–polyethylene glycol‐grafted polyethyleneimine copolymer were developed for the delivery of the cancer therapeutic, human tumor necrosis factor *α*‐related apoptosis‐inducing ligand, to glioblastoma and were able to cross the BBTB.^[^ ^115^ ^]^

## 3 Tunable Nanoparticle Properties for Enhanced BBB Transfer

Various factors can affect the performance of nanoparticles in BBB penetration such as surface ligands, charge, particle size, and shape. In this section, these features are discussed with an emphasis on polymeric nanoparticle systems.

### 3.1 Effect of Surface Ligands

The surface functionality of polymeric nanoparticles is the most important factor in BBB crossing efficiency. Specific ligands such as surfactants, antibodies, and peptides can be conjugated onto nanoparticles to promote recognition by receptors on the endothelial cells, leading to transcytosis and thus BBB crossing. Here, we provide an overview of the most promising ligands to enhance BBB penetration and their use in polymeric nanoparticles (**Table** **2**).

**Table 2:** An overview of ligands used to target the BBB, their specific targets, and examples of animal models and cell lines used

Ligands	Receptors	Used animal models and cell lines	References
**Surfactants**			
Polysorbate 80, Poloxamer 188	LDL receptor	Rats, mice Rat endothelial cells: RBE4 cells Bovine brain microvascular endothelial cell	^[^ ^116^, ^117^, ^118^, ^119^, ^120^, ^124^, ^125^, ^126^ ^]^
**Natural proteins**			
Lactoferrin	LRP receptor and lactoferrin receptor	Rats, mice	^[^ ^137^ ^]^
Melanotransferrin	Unknown receptor	Bovine brain capillary endothelial cell: BBCEC	^[^ ^139^ ^]^
Transferrin	Transferrin receptor	Human brain endothelial cell line: hCMEC/D3	^[^ ^138^ ^]^
Apolipoprotein	LDL receptor	Human brain endothelial cell line: hCMEC/D3	^[^ ^136^ ^]^
CRM197	Diphtheria toxin receptor	Mice	^[^ ^140^ ^]^
**Antibodies**			
OX26, RI7217, 8D3	Transferrin receptor	Rats, mice Brain microvascular endothelial cells: BMECs Brain capillary endothelial cells: BCECs	^[^ ^144^, ^145^, ^146^, ^149^ ^]^
83‐14 Mab, 29B4	Insulin receptor	Mice Human brain microvascular endothelial cells: HBMECs	^[^ ^147^, ^148^ ^]^
**Peptides**			
Angiopep‐2, Apolipoprotein E peptide	LRP receptor	Rats, mice Bovine brain capillary endothelial cell: BBCEC Rat endothelial cells: RBE4 cells Brain capillary endothelial cells: BCECs Mouse brain endothelial cells: bEnd.3	^[^ ^88^, ^157^, ^158^, ^159^, ^161^, ^162^ ^]^
Peptide T7, Peptide B6, THR, CGGGHKYLRW, CRT, miR9, PQVGHL, TPL, TAT	Transferrin receptor	Rats, mice Human brain capillary endothelial cells Mouse brain endothelial cells: bEnd.3 Bovine brain microvascular endothelial cells	^[^ ^54^, ^163^, ^164^, ^165^, ^166^, ^167^, ^168^, ^169^, ^175^ ^]^
Leptin30 peptide	Leptin receptor	Mice Brain capillary endothelial cells: BCECs	^[^ ^170^ ^]^
Glycopeptide G7	Unknown receptor	Mice	^[^ ^171^ ^]^
TGN, T‐T, TOL, CGN	Unknown receptor	Mice, Mouse brain endothelial cells: bEnd.3	^[^ ^172^ ^]^
Rabies virus glycoprotein peptide	Acetylcholine receptor	Mice Mouse brain endothelial cells: bEnd.3	^[^ ^78^ ^]^
**Aptamers**			
RNA‐based aptamer A15	Unknown receptor	Mice Mouse endothelial cell line: bEnd.3	^[^ ^183^ ^]^
DNA homologue ‐aptamer	Transferrin receptor	Mice	^[^ ^184^ ^]^
**Small molecules and other ligands**			
Maltobionic acid, glucose	GLUT receptor	Mice Mouse endothelial cell line: bEnd.3	^[^ ^82^, ^85^, ^185^ ^]^
l‐Glutathione	Glutathione receptor	Human brain endothelial cell line: hCMEC/D3	^[^ ^84^ ^]^
Adenosine	Adenosine G‐protein‐coupled receptors A2	Mice Mouse endothelial cell line: bEnd.3	^[^ ^172^, ^186^ ^]^

#### 3.1.1 Polysorbate 80

**Figure 8:**
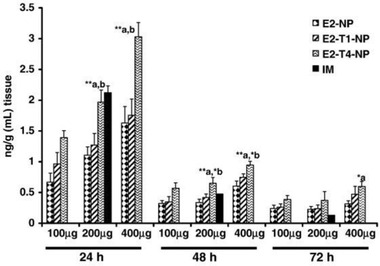
PS80 coated PLGA nanoparticles increase the brain delivery of estradiol after oral administration. E2‐NP: estradiol‐loaded PLGA nanoparticle; E2‐T1‐NP: estradiol with 1% PS80 coated PLGA NP; E2‐T4‐NP: estradiol with 4% PS80 coated PLGA NP; IM: intramuscular administration. Reproduced with permission.^[^ ^120^ ^]^ Copyright 2011, Elsevier.

Polysorbate 80 (PS80, Tween 80) is a common surfactant used to enable drugs to pass through the BBB^[^ ^116^ ^]^ and has been used for several pharmaceutical applications as an emulsifier. PS80 promotes BBB crossing due to the adsorption of apolipoprotein onto the nanoparticles, resulting in LDL receptor mediated transcytosis in epithelial BBB cells.^[^ ^117^ ^]^ Polysorbates are derived from ethoxylated sorbitan, which is esterified with fatty acids. Tröster et al. investigated several surfactants and demonstrated that polysorbate‐coated poly(methyl methacrylate) nanoparticles had an increased brain uptake.^[^ ^118^ ^]^ This was confirmed by other studies using polymeric nanoparticles with PS80 resulting in the highest CNS uptakes.^[^ ^119^ ^]^ The amount of PS80 on a particle was shown to be an important parameter too as 4% of PS80‐coated PLGA nanoparticles displayed a higher uptake in the brain of an Alzheimer's disease rat model than 1% PS80‐coated PLGA nanoparticles or non‐coated nanoparticles (**Figure** **8**).^[^ ^120^ ^]^

#### 3.1.2 Poloxamer 188

Poloxamers are non‐ionic triblock amphiphilic copolymers consisting of poly(ethylene oxide)‐poly(propylene oxide)‐poly(ethylene oxide).^[^ ^121^ ^]^ The first two digits (e.g., 18 in poloxamer 188) indicate the approximate molecular mass of the polypropylene core (1800 g mol^−1^) and the last digit (e.g., 8 in poloxamer 188) multiplied by 10 gives the percentage of polyoxyethylene content (80%).^[^ ^122^ ^]^ In particular, Poloxamer 188 has been used in the field of drug delivery and is approved by the FDA under the trade name of Pluronic F68.^[^ ^123^ ^]^ Similar to PS80, Poloxamer 188 coated nanoparticles promote the adsorption of apolipoprotein on the surface of nanoparticles in plasma leading to LDL receptor‐mediated transcytosis.^[^ ^124^ ^]^ Several polymeric nanoparticle systems have been successfully delivered to the brain using a poloxamer 188 coating, including PBCA^[^ ^124^ ^]^ and PLGA.^[^ ^125^ ^]^ In most studies, the BBB crossing efficiency for poloxamer 188 coating is similar to PS80, although the nanoparticle system seems to also have a minor influence.^[^ ^126^ ^]^

#### 3.1.3 PEG

PEG is approved by the FDA for human intravenous, oral, and dermal applications.^[^ ^127^ ^]^ Surface coating of nanoparticles with PEG results in a distinct reduction and modification of the protein corona that contains an abundance of clusterin proteins which limits non‐specific cell uptake,^[^ ^128^ ^]^ and therefore clearance by the MPS. This results in a better biocompatibility, longer circulation time, and decreased aggregation.^[^ ^129^ ^]^ Longer PEG chains (higher molecular weight), higher grafting densities, branched PEG chains, and methoxy termination can lead to longer circulation time.^[^ ^127^ ^]^ Although PEG coatings do not directly increase BBB penetration, the longer circulation time increases the probability of decorated ligands on nanoparticles to interact with BBB receptors. Therefore, many polymeric nanoparticle systems have been coated with PEG: for example, PCL,^[^ ^130^ ^]^ PLGA,^[^ ^131^ ^]^ PACA,^[^ ^132^ ^]^ chitosan,^[^ ^96^ ^]^ and PAMAM.^[^ ^133^ ^]^ Interestingly, the length of the PEG chain can also impact on polymeric nanoparticle penetration within the brain extracellular space. PLA nanoparticles (100 nm) coated with PEG chain lengths of 1 to 10 kDa and longer chain PEGs (5 and 10 kDa) resulted in the deepest brain parenchyma penetration.^[^ ^134^ ^]^ Other polymers with enhanced circulation time are: polysulfoxides, poly(glycerol)s, poly(amino acid)s, poly‐(vinylpyrrolidone), poly(2‐oxazoline)s, and poly(N‐(2‐hydroxypropyl)methacrylamide).^[^ ^135^ ^]^

#### 3.1.4 Natural Proteins

The natural ligands for receptors expressed on the BBB, such as the transferrin receptor, lipoprotein receptors, and diphtheria receptor, can also be used to improve the transcytosis of polymeric nanoparticles. Apolipoprotein,^[^ ^136^ ^]^ transferrin,^[^ ^137^ ^]^ lactotransferrin,^[^ ^138^ ^]^ and melanotransferrin^[^ ^139^ ^]^ have been successfully conjugated onto nanoparticles to improve BBB transcytosis. For example, PLGA nanoparticles coated with either transferrin or lactotransferrin showed a higher targeting efficacy (2.4 and 3.9 fold increase) in a mouse brain compared to non‐conjugated PLGA nanoparticles.^[^ ^138a^ ^]^ Moreover, PLGA nanoparticles modified with a mutated form of diphtheria toxin (CRM197) also exhibited an enhanced particle uptake.^[^ ^140^ ^]^ However, a potential disadvantage for this approach is that protein‐conjugated nanoparticles encounter competition from endogenous proteins. Furthermore, it is important to tune the avidity of the nanoparticles, namely, natural ligand density, as nanoparticles with a high surface density of ligands remain strongly attached to brain endothelial cells, whereas those with less proteins are capable of binding to the relevant protein receptor on the luminal side of the BBB and detaching from the receptor on the brain side.^[^ ^141^ ^]^ An alternative approach is to conjugate targeting ligands via responsive linkages to nanoparticles, which can be cleaved during transcytosis.^[^ ^142^ ^]^ Moreover, cationized proteins can be used to exploit adsorptive‐mediated transcytosis. For example, albumin‐coated nanoparticles did not increase BBB penetration, while cationic albumin‐coated nanoparticles exhibited a significantly higher BBB crossing.^[^ ^143^ ^]^

#### 3.1.5 Antibodies

**Figure 9:**
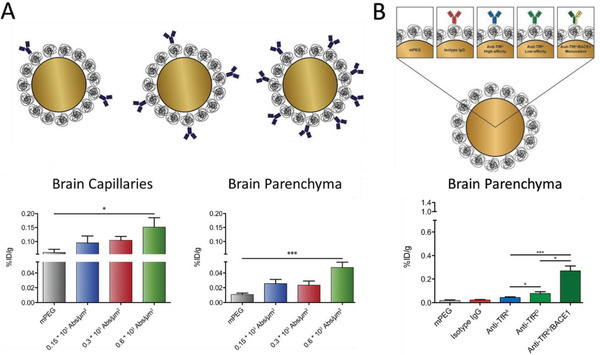
The effect of antibody density and affinity on the BBB penetration of nanoparticles. A) PEG‐coated gold nanoparticles with increasing low‐range transferrin antibody (RI7) surface densities showed increased brain uptake. Reproduced with permission.^[^ ^145^ ^]^ Copyright 2019, Elsevier. B) Transferrin receptor antibodies (TfR) with different affinities (anti‐TfR^A^ > anti‐TfR^D^, isotype lgG as control, and anti‐TfR^A^/BACE1 as bivalent antibody) were conjugated onto PEG‐coated gold nanoparticles and brain uptake was investigated. Reproduced with permission.^[^ ^153^ ^]^ Copyright 2018, Ivyspring International Publisher.

Antibodies recognize a unique motive in a receptor and are therefore well suited for receptor‐mediated transcytosis. Moreover, antibodies bind at different epitopes than endogenous ligands, such as proteins, preventing competitive binding. Therefore, antibodies have become a popular strategy to improve nanoparticulate brain delivery. Popularly used antibodies are the anti‐transferrin receptor antibodies (e.g., OX26,^[^ ^144^ ^]^ RI7217,^[^ ^145^ ^]^ and 8D3^[^ ^146^ ^]^) and anti‐human insulin receptor antibodies (83‐14 Mab^[^ ^147^ ^]^ and 29B4).^[^ ^148^ ^]^ For example, hyperbranched polyglycerol‐conjugated PLGA nanoparticles were functionalized with OX26 and loaded with endomorphins.^[^ ^149^ ^]^ In an in vivo model, these antibody‐modified nanoparticles showed a pronounced analgesic effect compared to nanoparticles without OX26.^[^ ^149^ ^]^ The antibody density on nanoparticles is also an important factor for the BBB penetration. For instance, the Moos group compared three low‐range densities of transferrin receptor antibodies (0.15, 0.3, and 0.6  x  10^3^ antibodies µm^−2^) conjugated onto gold nanoparticles and liposomes for BBB transport in both in vitro model and in mice (**Figure** **9**A).^[^ ^145^ ^]^ They discovered that nanoparticle systems with higher antibody density increased the BBB transport after intravenous administration. On the other hand, too much antibody coverage on nanoparticles can actually limit BBB crossing as the antibodies remain associated with the receptor.^[^ ^150^ ^]^ The brain uptake can also be improved by tuning the affinity of antibodies. Specifically, antibodies with high and low affinities mediate a low and intermediate uptake of nanoparticles into the brain, respectively, whereas a monovalent (bi‐specific) antibody with an intermediate affinity improved the uptake capacity remarkably (Figure 9B).^[^ ^145^, ^151^ ^]^

#### 3.1.6 Peptides

Peptides are short chains of amino acids that have shown great potential as conjugating ligands to promote BBB transcytosis due to their relatively low cost, reduced immunogenicity,^[^ ^152^ ^]^ and versatility for conjugation.^[^ ^153^ ^]^ BBB penetrating peptides, often called BBB shuttle peptides, are derived from neurotropic endogenous proteins, discovered by phage display or naturally occurring.^[^ ^153^ ^]^ Like antibodies, most peptides do not compete in binding with endogenous counterparts. However, their medium‐to‐low binding affinity to receptors promote nanoparticle release into the brain parenchyma compared to high affinity antibodies.^[^ ^153^, ^154^ ^]^ BBB shuttle peptides have been used extensively to increase brain delivery of small molecules, macromolecules, and nanoparticles.^[^ ^155^ ^]^

**Figure 10:**
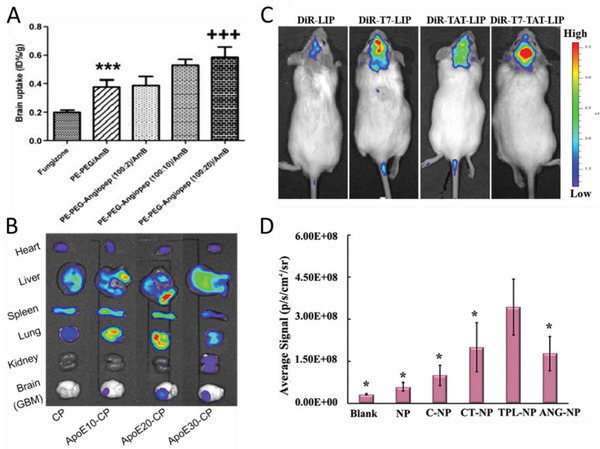
Effect of peptide ligands on the BBB penetration of nanoparticles. A) Biodistribution of free amphotericin B(AmB) and AmB‐incorporated micellar formulations with different ratios of angiopep‐2. Reproduced with permission.^[^ ^159^ ^]^ Copyright 2010, Elsevier. B) *Ex vivo* accumulation of chimeric polymersomes with varying ApoE peptide surface densities. Reproduced with Permission.^[^ ^88^ ^]^ Copyright 2018, American Chemical Society. C) Representative in vivo images of tumor‐bearing mice injected with dye (DiR) labeled liposomes (LIP) with T7‐peptide, TAT‐peptide and both T7‐peptide and TAT‐peptide. Reproduced with permission.^[^ ^163^ ^]^ Copyright 2014, American Chemical Society. D) The relative integrated fluorescence intensity in the brain of peptide conjugated nanoparticles: CGN peptide (C‐NP), CT peptide (CT‐NP), fusion peptide (TPL‐NP) or angiopep‐2 (ANG‐NP). Reproduced with permission.^[^ ^164^ ^]^ Copyright 2020, Elsevier.

The most used BBB shuttle peptide, angiopep‐2, consists of 19 amino acids and was derived from the Kunitz domain of aprotinin, an LRP1 and LRP2 ligand.^[^ ^156^ ^]^ Angiopep‐2 exhibits a higher transcytosis capacity than transferrin, lactoferrin, and avidin.^[^ ^157^ ^]^ It can easily be modified with cysteine using solid phase peptide synthesis to enable conjugation to maleimide or epoxide functional polymeric nanoparticles. For example, PEG‐PLA nanoparticles were functionalized with angiopep‐2 using this method and displayed an increased brain uptake in mice.^[^ ^157^, ^158^ ^]^ The angiopep‐2 density on nanoparticles also affects BBB penetration.^[^ ^159^ ^]^ 1,2‐Distearoyl‐sn‐glycero‐3‐phosphoethanolamine‐N‐[methoxy(polyethylene glycol)‐2000] (PE‐PEG) nanoparticles were prepared with a PE‐PEG to angiopep‐2 ratio of 100:2, 100:10, and 100:20. It was shown that a higher ratio (100:20) significantly increased the particle uptake in both brain capillary endothelial cells in vitro, as well as in a mouse brain (**Figure** **10**A). Other LDL receptor family targeting peptides have shown some promising results too.^[^ ^160^ ^]^ For example, apolipoprotein E peptide (ApoE) modified polymersomes had a 2.2‐fold higher BBB penetration in an endothelial cell monolayer compared to angiopep‐2 modified polymersomes.^[^ ^88^ ^]^ This may be ascribed to its high affinity to multiple LDL‐receptors, including LDLR, LRP1, and LRP2.^[^ ^161^ ^]^ The ApoE polymersomes also successfully delivered saporin to an orthotopic glioblastoma model resulting in complete tumor growth inhibition, although it must be stated that the BBTB is likely to have different penetration characteristics compared to a fully intact BBB (Figure 10B).^[^ ^88^ ^]^ A synergic approach has also been explored by modifying PEG‐PCL nanoparticles with both ApoE and PS80.^[^ ^162^ ^]^ Oral administration of this system resulted in an enhanced brain uptake of donepezil, an acetylcholinesterase inhibitor used for Alzheimer's disease.^[^ ^162^ ^]^

The transferrin receptor expressed on the endothelial cells of the BBB can be effectively targeted using BBB shuttle peptides. For example, the B6‐peptide was discovered by phage display and used to modify PEG‐PLA nanoparticles to enhance the brain delivery of a neuroprotective peptide.^[^ ^165^ ^]^ Furthermore, the THR peptide also showed to improve the brain uptake of gold nanoparticles in mice.^[^ ^166^ ^]^ Other transferrin receptor peptides that have been used for nanoparticle delivery are: CGGGHKYLRW,^[^ ^167^ ^]^ CRT,^[^ ^168^ ^]^ miR9,^[^ ^169^ ^]^ and PQVGHL.^[^ ^54^ ^]^ Peptide‐conjugated nanoparticles for targeting other receptors on the BBB have also been studied. This includes the leptin30 peptide to target the leptin receptor,^[^ ^170^ ^]^ glycopeptide G7 for an unknown receptor,^[^ ^171^ ^]^ TGN peptide for another unknown receptor,^[^ ^172^ ^]^ and rabies virus glycoprotein peptide (RVG) for the acetylcholine receptor.^[^ ^78^ ^]^ The latter enhanced DNA transport across the BBB using PEG‐PEI nanoparticles.^[^ ^173^ ^]^ However, RVG modified pH‐sensitive polymersomes could not pass the BBB in an in vitro assay in contrary to angiopep‐2 modified polymersomes.^[^ ^78^ ^]^ Glycopeptide and non‐toxic mutant of diphtheria toxin (CRM197) conjugated PLGA nanoparticles crossed the BBB to a similar extent, but higher than non‐peptide conjugated nanoparticles.^[^ ^140^ ^]^ Furthermore, the BBB shuttle peptides RVG, rabies virus matrix protein fragment (RVMAT), or TGN were compared for the brain delivery of 90 nm sized PLGA‐DSPE‐PEG nanoparticles, and only TGN peptide modified nanoparticles showed a slightly higher uptake than unmodified nanoparticles.^[^ ^172^ ^]^ Endogenous peptides have also been exploited. For example, insulin was conjugated onto human serum albumin nanoparticles using an NHS‐PEG‐maleimide linker and the loperamide‐loaded nanoparticles induced significant antinociceptive effects in the tail‐flick test in mice.^[^ ^174^ ^]^ Nevertheless, these natural peptide‐conjugated nanoparticles need to compete with endogenous peptides for binding, and additionally the use of insulin could potentially affect the regulation of glucose homeostasis.

A different class of peptides to promote BBB crossing are the cell penetrating peptides (CPPs). These peptides mainly consist of amphipathic or cationic sequence that is able to cross cellular membranes.^[^ ^153^ ^]^ Some common CPPs are model amphipathic peptide (MAP), transportan, antennapedia, and transactivator of transcription (TAT).^[^ ^175^ ^]^ For example TAT peptide conjugated PLA nanoparticles were shown to have enhanced brain uptake of ritonavir, a protease inhibitor, and prevented the efflux action of P‐gp.^[^ ^176^ ^]^ However, CCPs exhibit various levels of cytotoxicity.^[^ ^177^ ^]^ CPPs can also be combined with BBB shuttle peptides to enable dual‐targeting. For instance, TAT was combined with a transferrin receptor targeted peptide (T7) to enhance the transport of doxorubicin‐loaded liposomes in an BBB in vitro model and a glioma mouse model where T7 enhanced brain selectivity and TAT increased uptake in the brain tumor (Figure 10C).^[^ ^163^, ^166^ ^]^ An alternative approach to achieve dual‐targeting is to use the so‐called fusion peptides in which both peptides are linked. For instance, a fusion peptide named TPL was synthesized that linked the BBB shuttle peptide, TGN, and the neuron binding peptide, Tet_1_, via a four‐glycine linker. Fusion peptide modified PEG‐PLA nanoparticles showed a 5.7‐fold higher fluorescence intensity in the brain of mice compared to control nanoparticles (Figure 10D).^[^ ^164^ ^]^

#### 3.1.7 Aptamers

Aptamers are short single‐stranded chains of RNA or DNA oligonucleotides obtained via in vitro selection of randomized oligonucleotides using Systematic Evolution of Ligands by Exponential enrichment (SELEX) that bind to molecules such as peptides or proteins. Compared to antibodies, aptamers have a higher conformational stability and can reversibly refold to their native conformation.^[^ ^178^ ^]^ Therefore, aptamers have been widely used to target and diagnose brain diseases such as Alzheimer's disease,^[^ ^179^ ^]^ stroke,^[^ ^180^ ^]^ brain tumors,^[^ ^181^ ^]^ and Parkinson's disease.^[^ ^182^ ^]^ Moreover, aptamers to facilitate BBB penetration have been developed, such as the RNA‐based aptamer, A15,^[^ ^183^ ^]^ and a transferrin‐receptor aptamer liposomal delivery system exhibited a higher uptake in the rodent brain compared to control liposomes.^[^ ^184^ ^]^ However, more research is needed to fully explore the potential of aptamers to enhance BBB crossing of nanoparticles.

#### 3.1.8 Small Molecules and Other Ligands

Small molecules such as maltobionic acid,^[^ ^82^ ^]^ glutathione,^[^ ^84^ ^]^ glucose,^[^ ^82^, ^85^ ^]^ and natural polymers like chitosan have also shown to enhance the BBB and BBTB penetration of nanoparticles. Glucose‐conjugated nanoparticles can bind to the highly expressed BBB receptor GLUT‐1 to promote transcytosis with increased biocompatibility and tumor targeting. Similarly, 2‐deoxy‐d‐glucose modified poly(ethylene glycol)‐*co*‐poly(trimethylene carbonate) nanoparticles showed both enhanced BB(T)B crossing and uptake in glioma in mice,^[^ ^82^ ^]^ and as previously mentioned, several glucose‐modified polymeric micelle systems were developed and the effect of the glucose density on the particle surface was investigated by blending different ratios of polymers with/without glucose (Figure 6). They showed an optimal BBB penetration when approximately half of the copolymer strands were modified with glucose (Figure 6D).^[^ ^185^ ^]^ Interestingly, they were able to improve brain accumulation further by taking advantage of the rapid glycemic increase after fasting.^[^ ^85^ ^]^ In addition, adenosine‐modified polymeric nanoparticle systems consisting of a block copolymer of polylactic acid and hyperbranched polyglycerol (PLA‐HPG) were also studied, and nanoparticles with 10% adenosine showed a higher brain uptake than particles with 0, 1%, or 5% adenosine.^[^ ^172^ ^]^ Moreover, the 10% adenosine‐modified PLA‐HPG nanoparticles were compared to similar‐sized PLGA‐DSPE‐PEG nanoparticles conjugated with the BBB shuttle peptides RVG, RVMAT, or TGN, and exhibited a significantly higher brain uptake. However, it has to be noted that PLA‐HPG control nanoparticles showed a higher uptake than PLGA‐DSPE‐PEG nanoparticles. Adenosine is hypothesized to cross the BBB by carrier‐mediated transport through the concentrative nucleoside transporter type 2. Additionally, it also binds to G‐protein coupled receptor A2, producing a transient and controlled opening of the BBB.^[^ ^186^ ^]^

An alternative targeting ligand is chitosan which is often used as a stand‐alone nanoparticle system but can also be used as a surface coating as it is a cationic saccharide. For instance, chitosan‐modified PLGA nanoparticles loaded with carmustine showed a higher BBB penetration and anti‐tumor effect.^[^ ^187^ ^]^

### 3.2 Effect of Nanoparticle Size

The size of nanoparticles plays an important role in particle biodistribution, elimination, and CNS delivery.^[^ ^188^ ^]^ Polymeric nanoparticles can be administered via different routes, such as oral, intranasal, intravenous, or intraperitoneal. In particular, the intravenous injection method is explored as nanoparticles bypass the gastrointestinal tract and thus improve the bioavailability. Once intravenously administered, nanoparticles enter the cardiovascular system and can be transported across the BBB under suitable conditions. Prolonged circulation time is advantageous as it increases the probability of nanoparticles interacting with the BBB leading to subsequent transcytosis to the brain parenchyma.

Small molecules and nanoparticles with diameters less than 5 nm are rapidly cleared by the kidneys via glomerular filtration.^[^ ^189^ ^]^ Non‐continuous endothelia with vascular fenestrations are present in the liver, resulting in non‐specific accumulation of larger nanoparticles (50–100 nm). Furthermore, nanoparticles above 200 nm are retained in the spleen due to the 200–500 nm size range of splenic interendothelial cell slits.^[^ ^190^ ^]^ Microparticles in the size range of 2–5 µm tend to accumulate in the capillaries of the lungs. It is important to note that particle–cell surface interactions play an essential role in particle biodistribution as the MPS that is associated with the liver, spleen, and lungs can eliminate nanoparticles from the circulation by phagocytosis. In short, nanoparticles above 5 nm in size can escape the renal clearance resulting in a longer blood circulation time, but larger particles will eventually be cleared by the liver and the spleen. Furthermore, the biodegradation rate of nanoparticles also affects particle distribution as particles that degrade too rapidly could be cleared by the kidneys limiting their circulation time.

Polymeric nanoparticles can be synthesized to a specific size range^[^ ^191^ ^]^ and are therefore well suited to exploit the size effect on BBB interactions. For example, the effect of particle size on PLGA nanoparticle delivery in a brain injury model with a temporarily compromised BBB was studied.^[^ ^192^ ^]^ PEG‐coated PLGA nanoparticles with various sizes (100, 200, and 800 nm) were conjugated to a targeting ligand. The smallest size of nanoparticles (100 nm) showed a deeper penetration into the brain than the larger ones (800 nm). Furthermore, the liver retention of the largest PLGA nanoparticles was also double that of the smallest size of PLGA, indicating that the smaller particles had a longer circulation time. Similarly, it was shown that smaller PLGA nanoparticles (<100 nm) not only enhanced the ability of the particles to cross an intact BBB but also could penetrate into the brain parenchyma.^[^ ^193^ ^]^

The size effect (20 to 500 nm) was also investigated for polystyrene nanoparticles coated with d‐*α*‐tocopheryl polyethylene glycol 1000 succinate (PEG‐vitamin E) for BBB penetration in rats.^[^ ^194^ ^]^ It was observed that the smaller the nanoparticles the more pronounced the uptake into the brain (25 > 50 > 100 > 500 > 200 nm) and that the PEG‐vitamin E ligand was more effective in increasing BBB penetration for smaller particles than for larger particles. Interestingly, in a microfluidic model, a different non‐monotonic trend of particle dependence on size was observed where polystyrene particles of 200 nm in size exhibited the highest BBB transport than either smaller (100 nm) or larger (500 nm) particles.^[^ ^195^ ^]^ However, it must be noted that microfluidic models cannot accurately modulate the effects of the MPS. In contrast, some reports showed that BBB penetration had no effect on particle size. For example, 75 nm antibody‐PEG coated gold particles had a similar BBB penetration as 135 nm antibody‐PEG liposomes^[^ ^145^ ^]^ and the researchers concluded that particle size had no influence on BBB crossing.^[^ ^196^ ^]^ Here, PBCA nanoparticles were prepared in the range of 87 to 464 nm and coated with surfactants such as Lutrol‐SDS (non‐BBB‐passage) or polysorbate 80 (BBB‐passage). The barrier penetration was examined using the blood–retina barrier as a substitute model for BBB, which has similarities in transport and permeation. Nevertheless, there are clear cellular, structural, and functional differences between the blood–retina barrier and the BBB.^[^ ^197^ ^]^ Moreover, only limited quantitative analysis was performed and only a few sizes were investigated for each surfactant. However, the study did show that the nature of the surfactant was a more important determinant of uptake compare to particle size.

Apart from polymeric nanoparticles with a fixed size, size‐changeable nanoparticles have also been investigated to overcome multiple barriers including the BBB. An excellent example of this strategy is the earlier addressed study (Section 2.1.5), where 92 nm sized, cross‐linked telodendrimer micelles were able to cross the BBB, but were cleaved into smaller 14 nm secondary nanoparticles after exposure to the acidic tumor microenvironment to enhance transport to the brain tumor.^[^ ^82^ ^]^

### 3.3 Effect of Nanoparticle Shape

The most commonly explored nanoparticles in research have a spherical shape; however, nanoparticles can also be prepared with other shapes, for example, cubes, rods, discs, and stars. The shape of nanoparticles influences their behavior in blood flow, their interactions with endothelial cells, and the MPS, affecting circulation time, biodistribution, and cellular uptake.^[^ ^198^ ^]^ For example, filamentous micelles (diblock copolymer of PEG and poly(ethylethylene)) were shown to have longer circulation times^[^ ^199^ ^]^ than spherical micelles which could potentially be exploited to improve BBB interaction. This longer circulation time was also observed for PEGylated silica nanorods and was speculated to be aspect ratio dependent, which may be related to the uptake ability of the MPS^[^ ^200^ ^]^ and behavior in dynamic flow.^[^ ^201^ ^]^

**Figure 11:**
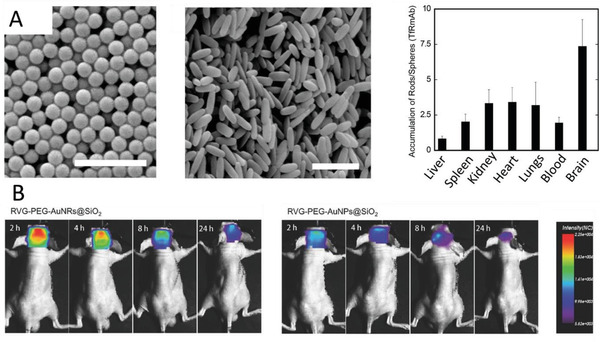
The effect of nanoparticle shape on BBB crossing. Scanning electron micrographs of transferrin antibody coated spherical and rod‐like nanoparticles, and their in vivo biodistribution (expressed as rods‐to‐spheres ratios). Scale bar 1 µm. Reproduced with permission.^[^ ^202^ ^]^ Copyright 2013, National Academy of Sciences. B) In vivo fluorescence signal image of rabies virus glycoprotein (RVG) peptide‐PEG‐coated silica‐gold nanorods (RVG‐PEG‐AuNRs@SiO_2_) and RVG peptide‐PEG‐coated silica‐gold nanospheres (RVG‐PEG‐AuNPs@SiO_2_) in orthotopic glioma xenograft‐bearing mice. Reproduced with permission.^[^ ^204^ ^]^ Copyright 2017, John Wiley & Sons.

The specificity of endothelial targeting can also be enhanced by engineering the shape of ligand‐displaying polymeric nanoparticles.^[^ ^202^ ^]^ Polystyrene nanospheres and nanorods with equivalent volumes were examined and their biodistribution in brain endothelium compared (**Figure** **11**A). The polymeric nanoparticles were conjugated to an anti‐transferrin receptor antibody and rod‐shaped nanoparticles showed a sevenfold higher accumulation in the brain compared to spherical particles, while some uptake enhancement (two to three times higher) was also observed in kidney, heart, and lung. Moreover, rod‐shaped PEGylated polystyrene nanoparticles decorated with a vascular adhesion molecule‐1 (VCAM‐1) antibody showed a higher cell uptake in both static and flow conditions compared to spherical nanoparticles. However, no particle uptake differences were observed in vivo. The low aspect ratio (2:1) used, protein corona formation, or the lower number of antibodies on the surface of the rod‐shape nanoparticles could be potential reasons for the discrepancy in the results.^[^ ^203^ ^]^ On the contrary, another study recently reported that that while rod‐shaped polystyrene nanoparticles exhibited lower affinity in a microfluidic model compared to spherical nanoparticles, they showed a higher BBB transport when normalized by endothelial association.^[^ ^195^ ^]^ Similar shape effects were also observed for inorganic hybrid nanoparticles with a polymer shell. For example, gold nanorods coated with PEG and RVG (a BBB shuttle peptide) exhibited a higher brain accumulation in mice than the corresponding spherical nanoparticles^[^ ^204^ ^]^ (Figure 11B). Other shapes such as nanodiscs, cubic nanocages, and nanostars are yet to be investigated for BBB penetration efficiency but have shown shape‐dependent biodistribution and cell association that could potentially also lead to enhanced BBB penetration. For instance, gold nanoparticles with a nanodisc shape were found to have higher tumor penetrations relative to nanorods and nanocubes in a tumor‐bearing mouse model.^[^ ^205^ ^]^ Furthermore, gold nanostars had a higher tumor uptake compared to nanospheres that was attributed to an increased surface contact.^[^ ^206^ ^]^

### 3.4 Effect of Surface Charge

The surface charge of polymeric nanoparticles can also affect BBB penetration.^[^ ^207^ ^]^ The luminal side of the BBB has a negative charge due to the presence of proteoglycans expressed on endothelial cells. As a consequence, positively charged nanoparticles can take advantage of adsorptive‐mediated transcytosis using either intrinsically positively charged nanoparticles^[^ ^77^ ^]^ or by conjugating positively charged ligands such as cationic albumin or cell binding peptides onto nanoparticles.^[^ ^208^ ^]^ Electrostatic interactions are triggered between the positively charged nanoparticles and negatively charged cell membrane, leading to endocytotic internalization of the nanoparticles.^[^ ^209^ ^]^ However, particles with cationic surfaces also exhibit a higher macrophage uptake and clearance by the MPS compared to neutral surface charge nanoparticles (e.g., PEG).^[^ ^210^ ^]^ Another potential drawback of using positively charged nanoparticles is that they may show a higher toxicity than anionic and neutral nanoparticles.^[^ ^209^ ^]^ Charged particles can also have a negative effect on BBB integrity. One study demonstrated that while neutral or low concentrations of anionic nanoparticles had no impact on the BBB integrity, higher concentrations of anionic and cationic nanoparticles both disrupted the BBB and had immediate toxic effects on brain microvasculature endothelium.^[^ ^211^ ^]^ The charge of nanoparticles also affects protein corona formation. While proteins are predominantly negatively charged, studies have shown that specific plasma proteins could also bind to some extent to various anionic and neutral nanoparticles,^[^ ^212^ ^]^ which also affects receptor binding and BBB transcytosis.^[^ ^213^ ^]^

## 4 Testing Models for BBB Transfer

### 4.1 In Vivo Models for BBB

Prior to clinical trials, in vivo models (e.g., rodent models) are employed to test the efficacy, immune response, and toxicity of drug‐loaded polymeric nanoparticles.^[^ ^214^ ^]^ Resulting data extracted from rodent models are often used to extrapolate to the human condition as comparable BBB permeability and the presence of efflux transporter proteins were reported in rodent and human brains.^[^ ^24^, ^215^ ^]^ Nanoparticle permeability and transport can be assessed using both invasive and non‐invasive techniques. Examples of invasive methods are compound permeation and product determination by intravenous injection and in situ brain perfusion, quantitative audiography, and microdialysis sampling.^[^ ^216^ ^]^ Examples of non‐invasive techniques are positron emission tomography (PET), single‐photon emission‐computed tomography (SPECT), and in vivo fluorescence imaging.^[^ ^216^ ^]^ Moreover, these imaging techniques, including magnetic resonance imaging (MRI), can also be used to assess disease progression.

The main challenge of drug delivery to the CNS is the rapid opsonization of the drug by the MPS. Different drug delivery routes, such as nasal, local, and systemic delivery have been developed in vivo aiming to transport drugs more efficiently to the brain.^[^ ^217^ ^]^ Systemic delivery is the most common technique as it transports the drug through carrier‐mediated endocytosis or efflux pump transporters. In some in vivo models, the integrity of the BBB can be temporarily disrupted using mannitol and focused ultrasound waves^[^ ^218^ ^]^ to deliver drugs into the CNS through the circulatory system. However, the intranasal delivery method is also gaining interest due to the rapid absorption of the drugs through the olfactory mucosa along the connective tissue surrounding the bundle of olfactory neurons.^[^ ^219^ ^]^ The intranasal delivery bypasses the BBB, which in some cases can be advantageous in drug delivery in CNS diseases.

In vivo models have an added advantage of pharmacokinetic (PK) and pharmacodynamics (PD) profiling of administered nanoparticles. PK is the study of the fate of the drug‐loaded nanoparticle in the body (including accumulation in the brain), whereas PD investigates the response of the body to the drug‐loaded nanoparticle and involves dose‐response relationships. In vivo models are capable of testing the nanoparticle penetration across the BBB and provide reliable PK/PD profiles. In PK studies, the nanoparticle concentration in the blood plasma is measured at several time points after administration. The most widely determined PK parameters are *C*_max_ (peak concentration), *T*_max_ (time to reach peak concentration), and half‐life (time taken to eliminate 50% of the drug). The total nanoparticle amount in the brain and in the blood plasma is determined using the area under the concentration‐time curve (AUC) that reflects the actual body exposure of the nanoparticle after administration. The average time that the drug remains in brain or plasma is given by the mean residence time (MRT). Other than quantifying PK parameters, the ability to examine the biodistribution of the nanoparticles in the body and especially in the CNS is important as it provides an insight on evasion of the MPS, and the penetration of the BBB and brain parenchyma. Systemic organs such as heart, liver, spleen, lungs, kidney, and brain are collected by sacrificing the animals at specific or several time points to analyze the drug content. Moreover, the organs provide information about the toxicity of the nanoparticles, ability of the nanoparticles to reach the site of action, release the drug payload, and the ability to achieve a therapeutic effect.

Drug concentrations in tissue extracts can be determined ex vivo using analytic techniques such as high‐performance liquid chromatography (HPLC) or enzyme‐linked immunosorbent assays (ELISA). For example, PK/PD profile of PLGA nanoparticles loaded with bevacizumab administered demonstrated higher brain availability in comparison to the free bevacizumab administered for 7 days as determined using ELISA.^[^ ^220^ ^]^ Similarly, HPLC studies of PEGylated albumin nanoparticles loaded with azidothymidine and decorated with transferrin demonstrated longer retention times in plasma and increased uptake in the brain compared to other organs.^[^ ^221^ ^]^ The plasma *AUC* and *MRT* of transferrin‐modified nanoparticles were significantly higher in comparison to free azidothymidine drug administered in mice. However, as these methods cannot be used to study polymeric nanoparticles directly, more recent approaches exploit fluorescent probes and radioisotopes to assess nanoparticle biodistribution. Moreover, these modalities can facilitate non‐invasive real‐time imaging.

#### 4.1.1 Fluorophore Labeling

**Figure 12:**
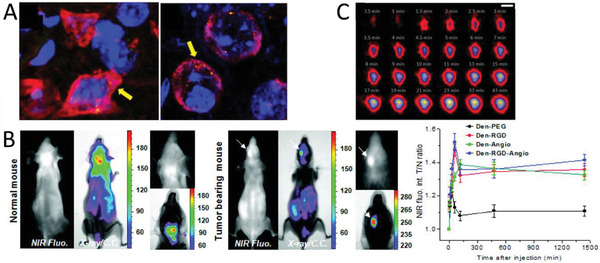
A) *Ex vivo* confocal microscopy images of the mouse neocortex showing the internalization of PLGA nanoparticles in neurons (yellow, arrows). Scale bar: 5 µm. Reproduced with permission.^[^ ^224^ ^]^ Copyright, 2017. B) Representative near infrared fluorescence and X‐ray/color coded fluorescence images of the normal mouse and brain tumor‐bearing mouse after injection of dendrimers labeled with the peptides RGD and angiopep‐2, and the in vivo time dependent NIR fluorescence tumor‐to‐normal (T/N) ratio. Reproduced with permission.^[^ ^225^ ^]^ Copyright 2012, American Chemistry Society. C) Coronal in vivo positron emission tomography images of the brain after the administration of PLGA nanoparticles labeled with the radioligand [^18^F]4‐fluorobenzylamide‐PEG_4_. Scale bar is 5 mm. Reproduced with permission.^[^ ^226^ ^]^ Copyright 2014, American Chemistry Society.

Fluorescence imaging has become one of the most popular imaging techniques in nanomedicine as it is highly sensitive, non‐toxic, and easy to incorporate. Fluorophores, such as fluorescein isothiocyanate (FITC), rhodamine, cyanines (Cy3 and Cy5), and coumarin are commonly used as imaging labels for polymeric nanoparticles.^[^ ^222^, ^223^ ^]^ Near infrared (NIR) dyes such as Cy5 have the added advantage of a deeper tissue penetration and limited auto‐fluorescence of tissue. Conventionally, fluorescence imaging requires sacrificing animals to observe the brain tissues through cryosection and histology. For example, FITC and rhodamine‐123 have been used to label PS80‐coated PBCA nanoparticles loaded with doxorubicin.^[^ ^222^ ^]^ These nanoparticles were injected into the tail vein and were used to study the time‐dependent distribution of the nanoparticles. Cryosection of brain tissues revealed that the nanoparticles were first observed in the capillary lumina and then localized in the endothelial cells of the brain capillary within 30 min. The nanoparticles distributed across the brain tissue within 1 to 2 h. FITC was also used to tag PLGA nanoparticles coated with ApoE peptide and lipocalin type prostaglandin‐d‐syntase.^[^ ^224^ ^]^ Brain sections were processed to determine the fate of intravenously administrated PLGA nanoparticles in mice using confocal microscopy (**Figure** **12**A). The nanoparticles were localized in the cerebral cortex parenchyma and the neurons and glial cells were demonstrated to internalize the nanoparticles with mild activation in astrocytes and glial cells.^[^ ^224^ ^]^ Furthermore, coumarin‐6 was used to conjugate to PLA nanoparticles modified with d‐*α*‐tocopheryl PEG succinate and transferrin.^[^ ^80^ ^]^ An ex vivo biodistribution study of the processed organs was carried out using HPLC with a fluorescence detector, and brain uptake was qualitatively assessed by means of confocal laser microscopy of brain tissue slices.^[^ ^80^ ^]^

The biodistribution of fluorophore‐labeled nanoparticles can also be investigated without sacrifycing the animal using real‐time non‐invasive fluorescence imaging systems. For example, poly(methyl methacrylate) nanoparticles loaded with rhodamine and highly fluorinated tetraphenylborate as bulky counterion displayed particularly bright fluorescence that enabled single‐particle tracking of these ultrabright fluorescent particles in the cerebral vessels using two‐photon intravital microscopy.^[^ ^227^ ^]^ Furthermore, the brain uptake of Cy5.5‐labeled dendrimers (Figure 12B)^[^ ^226^ ^]^ and NIR‐dye (DiR)‐labeled PEG‐PLA micelles^[^ ^228^ ^]^ were investigated using live animal imaging systems in vivo. Nevertheless, fluorescence imaging has several intrinsic limitations such as the limited penetration depth, tissue autofluorescence, and aggregation‐induced quenching, which makes this method less quantitative. In addition, fluorescent dyes have to be conjugated to the nanoparticles, which could potentially affect their PK/PD profile. Although NIR‐I dyes (650–950 nm) already have a deeper penetration depth,^[^ ^229^ ^]^ more improvements can be expected from novel NIR‐II dyes (1000–1700 nm) such as Ag_2_S that are able to explore deep‐tissues information in the range of 1 cm, and photoacoustic dyes can be imaged up to 5 to 10 cm deep into tissues.^[^ ^230^ ^]^

#### 4.1.2 Radioisotope Labeling

Other than fluorescent dyes, radioisotopes are also often used as imaging label agents due to their high sensitivity and unlimited penetration depth. However, handling of radioisotopes poses certain safety concerns over fluorescent dyes. The choice of radioisotope is also important as their half‐life determines their detection time in vivo. Either drugs or nanoparticles can be labeled with radioisotopes. Some examples of common radioisotopes used for labeling drugs are: ^125^I, ^3^H, and ^14^C. Metabolites derived from the radiolabeled drug molecules can be measured using radio‐HPLC techniques. For example, the amyloid affinity drug clioquinol was conjugated with ^125^I and was loaded into PBCA nanoparticles in a transgenic mice model for Alzheimer's disease.^[^ ^231^ ^]^ The biodistribution was assessed ex vivo using a gamma counter whereas in vivo storage phosphor autoradiography was also performed. Significant brain retention of the polymeric nanoparticles was observed in Alzheimer's disease transgenic mice compared to wild‐type mice.^[^ ^231^ ^]^

Nanoparticles can also be directly labeled with radioisotopes and can be tracked non‐invasively using techniques such as PET (for positron emitting radioisotopes) and SPECT (for photon emitting radioisotopes).^[^ ^232^ ^]^ PET offers the highest sensitivity and spatial resolution, but it is also more expensive.^[^ ^233^ ^]^ Furthermore, the biodistribution of radiolabeled nanoparticles in organs can also be measured ex vivo using a liquid scintillation counter or gamma‐counter. Lipid, inorganic, and polymeric nanoparticles can be engineered to chemically conjugate PET or SPECT radioisotopes, such as ^68^Ga, ^64^Cu, ^89^Zr, ^90^Y, and ^99m^Tc, through chelation or physical adsorption.^[^ ^234^ ^]^ Moreover, multiple radioisotopes can be attached to a single nanoparticle to enable multiplexing.^[^ ^235^ ^]^ Thus far, metallic nanoparticles, silica nanoparticles, liposomes, micelles, Q‐dots, dendrimers, and carbon nanotubes have been modified with various radioisotopes.^[^ ^234^ ^]^ For example, gold‐glyconanoparticles with a neuropeptide and chelator attached were radiolabeled with ^68^Ga to assess the biodistribution and BBB permeability in vivo using PET and ex vivo using a gamma counter.^[^ ^236^ ^]^ Intravenous administration of these nanoparticles in rats demonstrated accumulation of the particles in liver, spleen, and kidneys. Attachment of the targeting neuropeptide demonstrated 3‐fold higher distribution in brain and permeation into BBB in comparison to non‐targeted gold‐glyconanoparticles. In another example, PLGA nanoparticles with biotinylated radioligand, [^18^F]4‐fluorobenzylamide‐PEG_4_, were prepared for brain imaging in rodent models using PET (Figure 12C).^[^ ^225^ ^]^ The radiolabeled nanoparticles demonstrated delivery of the nanoparticles into the brain. PAMAM dendrimers were also designed with tumor vasculature targeting peptides and angiopep‐2.^[^ ^226^ ^]^ The nanoparticles were labeled with radioactive ^125^I to investigate the biodistribution and uptake in tumor‐bearing mice. In addition, PLGA nanoparticles radiolabeled with ^99m^Tc were delivered intranasally in rats.^[^ ^237^ ^]^ Blood and brain tissue samples collected post‐administration to evaluate the brain targeting efficiency via gamma scintigraphy imaging revealed higher uptake than free drug and localization of the nanoparticles in the brain after 30 min. Apart from polymeric nanoparticles, angiopep2‐functionalized multi‐walled carbon nanotubes were radiolabeled with ^111^In to evaluate the organ biodistribution in mice by *γ*‐scintigraphy.^[^ ^238^ ^]^

#### 4.1.3 Animal Behavioral Analysis

Evaluation of the nanoparticles/drug uptake to the brain can also be analyzed by behavioral tests in animal models. Drugs interfere with the brain signal and induce corresponding behavioral changes such as nociceptive response, motor function, or learning and memory alterations. The hot plate test and the tail‐flick tests measure the nociceptive response of an animal to a painful thermal stimulus and can be performed using healthy, wild‐type rodents. In these tests, the latency response time (typically between 10–60 s) to flick the tail, lick forepaws, or jump off the plate behavior is recorded. The percent maximum possible effect pre‐ and post‐drug latency is used to measure the efficacy of analgesics. For example, loperamide, an opiate receptor agonist that cannot cross the BBB easily, was encapsulated in PLGA nanoparticles to be used as a model drug to exert analgesic effects in mice. Intravenous injection of loperamide encapsulated in PLGA nanoparticles functionalized with 8D3 antibody showed an analgesic effect implicating the uptake of nanoparticles across the BBB in mice where the animals demonstrated instant antinociceptive activity in the tail‐flick test.^[^ ^146^ ^]^ Similarly, PBCA nanoparticles loaded with dalagrin and coated with apolipoproteins also demonstrated antinociceptive effects in mice within 30 min of administration.^[^ ^239^ ^]^

The locomotor and vegetative functions of the brain such as exploratory behavior, locomotor activity, and gait dynamics have also been investigated to demonstrate the uptake of polymeric nanoparticles into the brain.^[^ ^240^ ^]^ These behavioral tests are often used to investigate a disease, such as drug‐induced Parkinson's syndrome. For example, PS‐80 coated PBCA nanoparticles loaded with nerve growth factor were administered in a drug‐induced Parkinson's disease rat model,^[^ ^240^ ^]^ and a reduction in Parkinson's symptoms such as orientation‐research reaction, rigidity and tremor, and locomotor activity were observed. Moreover, the enhanced nerve growth factor concentrations in the brain tissue of mice measured using ELISA demonstrated the efficient transport of these polymeric nanoparticles across the BBB.

In conclusion, various approaches (e.g., fluorescent tags and radiolabeling) are used to assess the biodistribution, PK/PD profiling, and brain uptake of nanoparticle drug complexes in in vivo models. Each technique has its own advantages and disadvantages that need to be considered while designing experiments. Parameters such as administration route and sampling time points are crucial for an in vivo study. This allows researchers to tailor nanoparticles to increase circulation time, limit uptake by the MPS, and maximize brain uptake. The therapeutic effect can be demonstrated by animal behavior tests or using medical imaging modalities (e.g., MRI, PET/CT) to assess, for example, tumor reduction in brain cancers or reduction of *β*‐amyloid plaques in Alzheimer's disease. However, even when nanoparticles are successful in crossing the BBB, the in vivo fate of the nanoparticles in the brain, such as diffusion through the brain parenchyma or intracellular uptake, remains uncertain due to limited spatial resolution and the BBB transcytosis pathway cannot be studied. Therefore, intracellular mechanisms and trafficking of nanoparticles need to be studied in vitro. Moreover, animal models are expensive to establish, can be very labor intensive, and involve ethical concerns.

### 4.2 In Vitro Models

It was estimated that 50% of the responses from laboratory animals do not directly translate to human conditions.^[^ ^241^ ^]^ The complex human physiology and variation in the response of transporter proteins from species‐to‐species cannot be faithfully replicated in animal models.^[^ ^241^ ^]^ The in vivo studies require a substantial number of model organisms for drug screening, which involves increased cost, skilled personnel, and longer time to perform tests. There is hence an urgent need for more “humanized” in vitro BBB models. In comparison to animal models for representing the BBB, in vitro BBB models simulate quasi‐physiological models that can incorporate vascular hemodynamics and stimuli to induce the pathological conditions of neurodegenerative diseases (Table 1). In vitro BBB models offer advantages of non‐invasive tests such as permeability assays, trans‐endothelial electrical measurements, and high‐resolution microscopy imaging. These BBB models can be tailored for different configurations of cells to be cultured on 2D or 3D condition and can utilize different types of cells (e.g., cell line vs stem cells). Various in vitro models have been designed to mimic the in vivo patho(physio)logical state of the BBB, such as the membrane integrated static cell culture system (Transwell model), and shear stress inclusive models (e.g., dynamic in vitro model and microfluidic model). These in vitro models have been extensively exploited in screening nanoparticle/drug conjugates to study the BBB transfer efficiency.

#### 4.2.1 Quality Controls for In Vitro BBB Formation

Permeability measurements quantify the “leakiness” of the formed BBB in vitro. It helps to understand the passage of molecules across the BBB in drug discovery and drug screening studies. The permeability is commonly assessed using various fluorescent tracers, such as FITC‐labeled dextran molecules (4, 20, 50, 70 kDa), sodium fluorescein (376 Da), and Lucifer yellow (457 Da). The permeability coefficient of an analyte is determined by injecting the fluorophores and fluorophore labeled molecules through the apical side of the BBB, and quantifying the permeated molecules in the basolateral side over time.^[^ ^216^ ^]^ The measured or apparent permeability coefficient *P*_meas_ (cm s^−1^) is determined by Equation (1).^[^ ^242^ ^]^

(1) $$P_{\mathrm{meas}}=\;\;\frac{m_{a}}{A\;\left(C_{l}-C_{b}\right)}=\;\frac{QC_{b\;}}{A\;\left(C_{l}-C_{b}\right)}$$

where *A* (cm^2^) is the surface area in the selected region of interest, *Q* is the flow rate (µL min^−1^), and *m_a_* (mol s^−1^) is the mass transport rate of analyte across the membrane. *C_l_* (mol mL^−1^) and *C_b_* (mol mL^−1^) are the luminal and basal concentration of the analyte, respectively.

The permeability coefficient of the endothelial monolayer, *P*_endo_ (cm s^−1^) is then calculated by deducting the permeability coefficient measured in in vitro BBB systems without endothelium (*P*_blank_) from the measured permeability coefficient (*P*_meas_) by Equation (2).

(2) $$\frac{1}{P_{\mathrm{endo}}}=\;\frac{1}{P_{\mathrm{meas}}}-\frac{1}{P_{\mathrm{blank}}}$$

In vivo permeability coefficients have been obtained in animal models. For example, in rat's brain, the permeability coefficient for 4 kDa dextran is 6.2 × 10^−7^ cm s^−1^,^[^ ^243^ ^]^ while small molecule dyes such as sodium fluorescein (376 Da) and Lucifer yellow (457 Da) have permeability coefficients of 1.46 × 10^−6[^ ^244^ ^]^ and 1.5 × 10^−7^ cm s^−1^,^[^ ^245^ ^]^ respectively. Similar measurements have also been carried out for a wide variety of drugs, including diazepam, amitriptyline, and bupropion.^[^ ^246^ ^]^ In vitro BBB models can sometimes be created with permeability constants in a similar range to in vivo conditions; however, the majority of the permeability values reported are considerably higher. For example, 4.5 × 10^−6^ cm s^−1^ for sodium fluorescein, 3.2 × 10^−5^ cm s^−1^ for Lucifer yellow, and 1.0 × 10^−5^ cm s^−1^ for FITC‐dextran (4 kDa).^[^ ^247^ ^]^ Furthermore, a strong in vitro*/*in vivo correlation for hydrophilic drugs was demonstrated.^[^ ^246^ ^]^

The trans‐endothelial electrical resistance (TEER) is the gold standard technique to assess the integrity of the formed BBB in vitro through the use of electrodes.^[^ ^248^ ^]^ An alternating current voltage signal is applied with a square wave form across the monolayer of endothelial cells. TEER measures the resistance generated by the endothelial monolayer *R*_Tissue_ (Ω) which is inversely proportional to the effective area of the monolayer and is calculated using Equation (3).

(3) $$R_{\mathrm{Tissue}}=\;R_{\mathrm{Total}}\;-\;R_{\mathrm{Blank}}$$

where *R*_Blank_ (Ω) is the resistance of the system in the absence of cells and *R*_Total_ (Ω) is the total resistance measured in the presence of endothelial cells. The final TEER (Ω^.^cm^2^) is calculated using Equation (4).

(4) $$\mathrm{TEER}\;=\;R_{\mathrm{Tissue}}\times \mathrm{Area}$$

where, Area is given by the total area of the endothelium cell layer.

A TEER value of at least 150 Ω·cm^2^ is considered a reasonable value for endothelial cells cultured in static in vitro BBB models, whereas the in vivo TEER value is typically 1500–2000 Ω·cm^2^.^[^ ^249^ ^]^ High TEER values suggest increased upregulation of the tight junctions between the endothelial cells, which implicates restricted passive diffusion across the BBB. In fact, with sophisticated engineering of the in vitro model design system and choice of cell lines, some reports have achieved higher TEER values (from 1100 to 1300 Ω·cm^2^) close to that of estimated in vivo values.^[^ ^250^ ^]^ A recent review has tabulated the endothelial cell lines based on their TEER values, protein, and mRNA values.^[^ ^248^ ^]^ The TEER values for endothelial cells can vary drastically, which depends on factors such as cell source, culture duration, temperature, cell culture medium composition, co‐culture of endothelial cells in contact or non‐contact position with other brain cell types, and shear stress. An estimated of 36 immortalized cell lines are used in BBB research, of which the human‐derived cerebral microvascular endothelial cell line (hCMEC/D3), the rat endothelial cell line (RBE4), and the mouse brain microvascular endothelial cell line (bEnd.3) have been identified to closely resemble human endothelial cells.^[^ ^250^ ^]^ The human‐derived hCMEC/D3 endothelial cell line in particular preserves the in vivo characteristics of endothelial cells by expressing transporter proteins and receptors such as P‐gp, MDR‐1, BRCP, TfR, and other metabolizing enzymes that are used to study drug transport. It also demonstrates the topographical distribution of the tight junction proteins that are present within the endothelial cells. Similarly, bEnd.3 cell line also shows high expression level of tight junction proteins and demonstrates low paracellular permeability. In vitro models of the BBB are broadly classified into static and dynamic models, and microfluidic models.

#### 4.2.2 Static Transwell In Vitro Model

**Figure 13:**
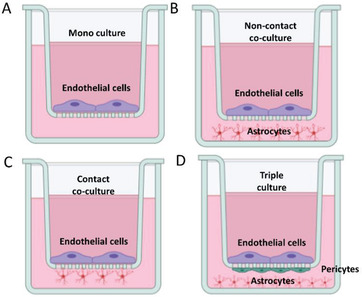
Static monolayer BBB model that uses a Transwell insert placed into a well plate. A) The endothelial cells are cultured on the top of the Transwell insert coated with extracellular matrix protein. 2D static Transwell models showing B) non‐contact co‐culture, C) contact co‐culture and D) triple culture with other brain cells such as astrocytes and pericytes.

A static model of BBB typically uses a Transwell insert setup consisting a porous polycarbonate membrane (typically 10 µm thick) with a pore size ranging from 0.4–8 µm. The endothelial cells are seeded on the porous membrane to form a BBB monolayer in the upper compartment. The Transwell setup can also include co‐culture with astrocytes and/or pericytes seeded on the other compartment (**Figure** **13**). The porous membrane allows the exchange of soluble factors and nutrients between the two compartments. Other porous membranes, such as PCL, polyester, and ultra‐thin silicon nitride (1 µm thickness) have also been used to develop static 2D BBB models.^[^ ^251^ ^]^ Porous membranes are typically coated with a layer of extracellular matrix proteins, such as collagen, Matrigel, or fibrinogen to facilitate cell adhesion, spreading, growth, and migration. The cell–matrix interaction influences cell–cell adhesion and the overall tissue structure. Furthermore, electrospun nanofibrous mats have been used to facilitate the alignment and orientation of the endothelial cells.^[^ ^252^ ^]^ In comparison to the porous polycarbonate membranes, a less stiff substrate such as electrospun mats or a collagen/Matrigel coated porous membranes modifies the arrangement of the actin bundles to enhance the interaction of tight junction proteins.^[^ ^253^ ^]^ This enables the regulation of endothelial monolayer integrity. Thus far, mono‐culture, bi‐culture, and tri‐culture static models using the Transwell system have been developed for modeling the BBB.

Mono‐culture static models are simple, inexpensive, and can allow high‐throughput drug screening (Figure 13A). For examples, mono‐culture Transwell models have been extensively used to determine and quantify transcytosis of transferrin‐conjugated gold, silica, titanium dioxide, and albumin nanoparticles.^[^ ^254^ ^]^ Kinetic rate parameters of poly‐[triphenylamine‐4‐vinyl‐(p‐methoxy‐benzene)] based polymeric nanoparticles using monolayer of bEnd.3 cell in a Transwell setup demonstrated that endocytosis of nanoparticles was faster compared to exocytosis of nanoparticles from the endothelial cells.^[^ ^255^ ^]^ In a separate study, bEnd.3 cells were cultured in Transwell inserts and treated with copolymer of PEG‐*b*‐poly(d,l‐lactide) nanoparticles loaded with curcumin and were shown to protect the endothelial cells against oxidative stress by scavenging of free radicals.^[^ ^256^ ^]^ A monolayer of brain capillary endothelial cells (BCECs) was treated with PEG‐*co*‐PCL nanoparticles decorated with angiopep‐2 to investigate the mechanism of nanoparticle uptake by endothelial cells.^[^ ^257^ ^]^ The nanoparticles were endocytosed through caveolae and clathrin‐mediated pathways involving a time‐, energy‐, and concentration‐dependent mode. However, recent studies also showed that nanoparticles could adhere to the insert membrane or the transport of nanoparticles was restricted due to the formation of multilayers of endothelial cells.^[^ ^258^ ^]^ To avoid such issues, a filter‐free in vitro BBB model was established with a monolayer of endothelial cells cultured on thick collagen gels to demonstrate the transcellular and paracellular transport of polymersomes.^[^ ^259^ ^]^ High‐throughput quantitative fluorescence spectroscopy measurement demonstrated 6.6% transcytosis of PEG‐*b*‐polybutadiene polymersomes decorated with G23 peptide across the BBB.^[^ ^259^ ^]^ The absence of co‐culture in this filter‐free BBB model resulted in low TEER values (100 Ω·cm^2^).

In co‐culture static models, a monolayer of endothelial cells is typically seeded on the apical (luminal) side of the membrane, and the astrocytes (or pericytes) are seeded on the basolateral (abluminal) side of the membrane. Co‐culture of endothelial cells with pericytes or astrocytes (bi‐culture) occurs either in non‐contact (Figure 13B) or in contact (Figure 13C) configuration. Co‐culture of endothelial cells with pericytes has shown enhanced upregulation of efflux pump protein P‐gp.^[^ ^260^ ^]^ On the other hand, co‐culture of endothelial cells with astrocytes in non‐contact culture enhanced the expression of the tight junction proteins, claudin‐5 and ZO‐1.^[^ ^260^ ^]^ The soluble factors secreted from astrocytes have shown to upregulate the expression of Glut‐1, Mdr1a, and Bcrp in endothelial cells.^[^ ^260^ ^]^ TEER values have been shown to be increased when endothelial cells are co‐cultured with astrocytes in non‐contact culture (< 500 Ω·cm^2^)^[^ ^261^ ^]^ in comparison to contact culture (55–297 Ω·cm^2^)^[^ ^262^ ^]^ and mono‐culture (80‐100 Ω·cm^2^). Various bi‐culture Transwell models have been employed to study polymeric nanoparticles crossing the BBB. For example, a bi‐culture Transwell set up with rat brain endothelial cells and rat astrocytes was developed to study the translocation of poly(mPEG_2000_cyanoacrylate‐*co*‐hexadecylcyanoacrylate) nanoparticles.^[^ ^263^ ^]^ The endothelial cells demonstrated significant uptake and localization of PEGylated nanoparticles in the cytoplasm and cell nucleus. Various other bi‐culture models were used to study the efficacy of polymeric nanoparticles to cross the BBB to target glioma cells. For example, a bi‐culture Transwell setup with BCECs and U87 glioma cells was used to examine the extent of cellular uptake of coumarin‐loaded PCL nanoparticles conjugated with different amounts of transferrin. The study further emphasized that the transferrin surface density affects the uptake of nanoparticles.^[^ ^264^ ^]^ In another study, docetaxel‐loaded PEG‐PLA nanoparticles were investigated for cellular uptake in RBE4/C6 rat astrocytoma contact‐based Transwell bi‐culture set up.^[^ ^265^ ^]^ The permeation of glutathione‐coated nanoparticles was greater than free docetaxel. Furthermore, a bi‐culture, non‐contact Transwell set up consisting of monolayer of human brain capillary endothelial cells on the insert and bovine pericytes were seeded in the opposite compartment to evaluate translocation of the cell penetrating peptides across the BBB.^[^ ^155a^ ^]^

More complex models have been established to incorporate both pericytes and astrocytes together with endothelial cells in a tri‐culture. Significant increase in TEER values in tri‐culture static BBB models were reported compared to mono‐culture models.^[^ ^260^, ^266^ ^]^ A tri‐culture with endothelial cells in contact with astrocytes and pericytes or neurons in a non‐contact position have also been developed (Figure 13D).^[^ ^267^ ^]^ For example, a Transwell setup involving complex co‐culture of brain capillary endothelial cells on one compartment of the porous membrane and a mixture of glial cells (60% astrocytes and 20% oligodendrocytes and 20% microglia) were seeded on the bottom of the well and was used to investigate the uptake of PLGA nanoparticles conjugated with transferrin.^[^ ^268^ ^]^ PLGA nanoparticles were demonstrated to be endocytosed by the endothelial cells through the receptor‐mediated endocytosis pathway. Usually, the endothelial cells endocytose the nanoparticles using an energy‐dependent process through clathrin pathway.^[^ ^269^ ^]^ However, inhibiting certain molecules involved in the clathrin‐ and caveolae‐pathway demonstrated that the nanoparticles were internalized by the endothelial cells through caveolae‐mediated transcytosis.

#### 4.2.3 Dynamic BBB Models

The absence of mechanical stimuli, such as shear stress (in the case of static Transwell system), limits the ability of culturing cells and maintaining the BBB over a longer duration. Endothelial cells when exposed to shear flow demonstrated enhanced cell differentiation, proliferation, and cell cycle regulation.^[^ ^260^ ^]^ Increased TEER were reported from a typical value of 70 Ω^.^cm^2^ in static models to 700 Ω·cm^2^ when cells were exposed to flow.^[^ ^270^ ^]^ Dynamic models that incorporate shear stress to mimic the physiological microenvironment of the BBB include the cone‐plate apparatus, dynamic in vitro (DIV), and microfluidic models.

**Figure 14:**
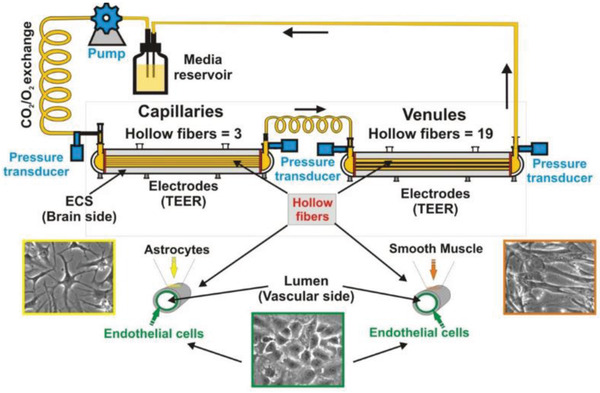
Dynamic in vitro BBB model connected to peristaltic pump that is used to induce shear stress. Reproduced with permission.^[^ ^275^ ^]^ Copyright 2018, Elsevier.

The cone–plate apparatus uses a viscometer setup to generate shear stress.^[^ ^271^ ^]^ A monolayer of endothelial cells grown on the plate is placed between a cone and a plate. The cone is rotated at an angle to generate shear stress to the system. However, the shear stress generated and experienced by the monolayer of endothelial cells is non‐uniform which limits its application.^[^ ^271^ ^]^ The DIV model uses porous hollow fibers (1 mm in diameter and 4.2 cm in length) inside a sealed chamber where the endothelial cells and astrocytes are seeded on the apical and basolateral side of the fibrous membrane, respectively (**Figure** **14**).^[^ ^272^ ^]^ The system is connected to two tubes: one for culture medium and another for gas exchange. Using a pulsatile pump apparatus, fresh culture media is pumped into the system at a flow rate from 1 to 50 mL min^−1^ to generate a corresponding shear stress of 5–23 dynes cm^−2^, which resembles the physiological levels of shear stress in brain capillaries. A further modification by incorporating transmural microholes (2–4 µm) in a porous hollow fibrous membrane demonstrates the extravasation of monocytes and a significant increase in the production of proinflammatory cytokines in the endothelial monolayer cultured at 4 dynes cm^−2^.^[^ ^273^ ^]^ However, the design of DIV‐BBB model has certain limitations, such as considerably large diameter of endothelial lumen in comparison to in vivo brain capillaries and inability of direct visualization of the intraluminal compartment to assess the changes in endothelial cell morphology (or nanoparticle uptake).^[^ ^274^ ^]^ In addition, this system requires 9 to 12 days to attain steady‐state TEER measurements, which prevents the use of such system as a dynamic BBB model in large‐scale screening settings.

Microfluidic models are developed by using microfabrication techniques to achieve a quasi‐physiological state of the BBB with respect to geometry, biochemical, and mechanical factors. The most common material to fabricate microfluidic models is polydimethylsiloxane (PDMS). PDMS is biocompatible, allows gas exchange, and is transparent, which facilitates direct visualization of cells at high resolution by microscopy. Microfluidic models have an advantage over aforementioned models, due to the potential to be used as high‐throughput screening in drug discovery studies. Microfluidic BBB chip models mimic the physiological in vivo microenvironment and accurately predict the permeability of the testing drugs. Some microfluidic models have been able to mimic pathological conditions such as cerebral ischemia^[^ ^276^ ^]^ and motor neuron disease.^[^ ^277^ ^]^ Although those models vary in design, they have a set of common features: a monolayer of cerebral endothelial cells cultured either on a plane or on cylindrical surface, an appropriate extracellular matrix for seeding the endothelial cells, and cross‐talk between endothelial cells and other brain cell types (e.g., astrocytes, neurons). Exposure of shear stress to endothelial cells, incorporation of electrodes to measure the TEER, and co‐culturing of astrocytes and neurons in 3D hydrogel are additional features explored in microfluidic BBB models.

Microfluidic BBB model design can vary in different complexity: dynamic 2D, 2.5D, and 3D configuration.^[^ ^278^ ^]^ In 2D microfluidic models, the endothelial cells are cultured on a flat, porous membrane. Astrocytes or pericytes are cultured on the opposite side of the membrane. In 2.5D model, the endothelial cells are cultured on a dense matrix to form a hollow lumen which is similar to the alignment of the cells in the blood vessels. The 3D model has two unique features: a hollow lumen of endothelial cells with astrocytes and neurons cultured in a hydrogel matrix to form a complete neurovascular unit. BBB microfluidic models have mimicked a number of physiological features such as, the luminal fluid shear stress flow,^[^ ^279^ ^]^ the interstitial fluid flow,^[^ ^280^ ^]^ cyclic strain,^[^ ^255a^ ^]^ the hollow and circular lumen that mimics the geometry of blood vessels,^[^ ^281^ ^]^ the 3D extracellular matrix for endothelial cells and for neuronal cells,^[^ ^282^ ^]^ the optimized fluid‐to‐tissue ratio,^[^ ^283^ ^]^ and the co‐culture with other brain cell types to form an entire neurovascular unit.^[^ ^247^, ^284^ ^]^

#### 4.2.4 2D Microfluidic Models

**Figure 15:**
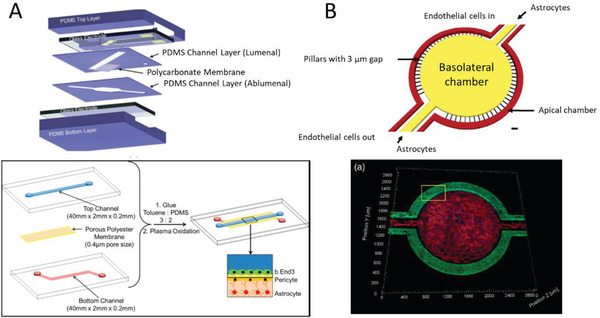
Dynamic 2D microfluidic BBB models. (A) PDMS sandwich models where the bilayer of PDMS is separated by either a polycarbonate^[^ ^286^ ^]^ or a porous polyester^[^ ^283^ ^]^ membrane. The endothelial cells are seeded on top side and pericytes are seeded on the bottom side of the membrane. The astrocytes are seeded at the bottom of the microchannel. Reproduced with permission.^[^ ^286^ ^]^ Copyright 2012, Royal Society of Chemistry. Reproduced with permission.^[^ ^283^ ^]^ Copyright 2016, American Chemical Society. B) PDMS parallel model showing apical and the basolateral chamber are separated by an array of pillars of the diameter 3 µm. Reproduced with permission.^[^ ^287^ ^]^ Copyright 2019, John Wiley & Sons.

2D microfluidic models follow a similar design to the Transwell apparatus, with a porous membrane sandwiched by two layers of PDMS channels, also referred to as the “sandwich” model (**Figure** **15**A).^[^ ^281a^ ^]^ Endothelial cells are seeded on the porous membrane, and in some cases, astrocytes are cultured on the other side of the membrane (contact culture). Electrodes are incorporated at the top and bottom of the PDMS chips enabling the TEER measurements.^[^ ^285^ ^]^ The co‐culture of astrocytes or astrocyte‐conditioned medium and shear stress in the range of 0.02–5 dynes cm^−2^ increased the BBB integrity with enhanced TEER values of 120–250 Ω·cm^2^.^[^ ^285^, ^286^ ^]^

The PDMS “sandwich” BBB model has been used to investigate different types of nanoparticles for their BBB transfer efficacy. For example, a microfluidic device comprised of two S‐shaped microchannels was used in which the overlapping region was separated by a porous polycarbonate membrane.^[^ ^288^ ^]^ Endothelial cells were seeded on the upper side of the membrane and were treated with liposome nanoparticles conjugated to angiopep‐2 under both static and flow conditions. At low shear stress (1 dyn cm^−2^), a high binding efficiency and successful transcytosis was observed, while at higher shear stress (6 dynes cm^−2^), the nanoparticle binding efficiency was reduced. In another study, human brain microvascular endothelial cells (HBMECs) were co‐cultured with pericytes and astrocytes using such “sandwich” system.^[^ ^289^ ^]^ Lipid‐mimetic nanoparticles conjugated with apolipoprotein A1 were used to investigate the particle uptakes by the endothelium. It was observed that the uptake of these nanoparticles was mediated by the scavenger receptor class B type I transporter protein.^[^ ^289^ ^]^ In another PDMS “sandwich” model, an ultrathin silicon membrane (50 nm thick and 0.5 µm pore size) was used for enhancing the imaging capability of observing nanoparticle uptake by the hCMEC/D3 endothelial cells.^[^ ^290^ ^]^ The ultrathin membrane enhanced the cell‐to‐cell contact between the endothelial cells and astrocytes. Smaller sized carboxylate‐modified polystyrene nanoparticles (40 nm) could translocate across the BBB more effectively than larger particles (100 nm). The authors also reported that ApoE peptide conjugated SiO_2_ nanoparticles demonstrated higher particle uptake compared to polystyrene nanoparticles due to the presence of ApoE peptide. This microfluidic device enabled real‐time visualization of the translocation events. Translocation events such as subcellular location of nanoparticles, increased lysosomal accumulation of nanoparticles in the endothelial cells, and colocalization of nanoparticles with lysosomes in the astrocytes clearly demonstrated the process of nanoparticles after uptake by endothelial cells.

Apart from PDMS, poly(methylmethacrylate) have been used to fabricate sandwich model of BBB with a polyester membrane of 3 µm pore.^[^ ^255c^ ^]^ Polystyrene nanoparticles were conjugated with membranotropic peptide, gH625, to facilitate the transport of nanoparticles across the BBB.^[^ ^255c^ ^]^ Under fluid flow condition, the nanoparticles were internalized by the endothelial cells, resulting in higher uptake and transport efficiency in comparison to non‐functionalized polystyrene nanoparticles. Also, membrane‐less PDMS parallel 2D models have been explored with the easier fabrication process and compatible for high‐resolution imaging. For example, a microfluidic model with two side‐by‐side circular chambers separated by an array of pillars (with 3 µm gaps) was developed (Figure 15B).^[^ ^291^ ^]^ Under continuous perfusion of media for 4 days and in the presence of astrocyte‐conditioned medium, barrier tightness of monolayer of RBE4 endothelial cells was increased. Upregulation of the P‐gp efflux transporter protein was also reported.

#### 4.2.5 2.5D Microfluidic Models

**Figure 16:**
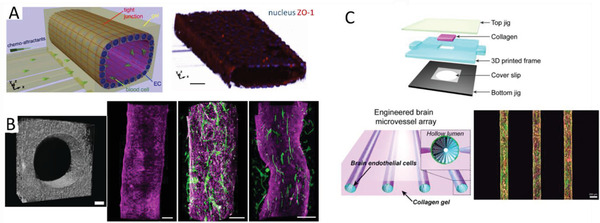
Examples of dynamic 2.5D microfluidic BBB models. A) Functionalization of the PDMS microchannel using poly‐L‐lysine to form a circular endothelial lumen. Scale bar 50 µm. Reproduced with permission.^[^ ^276^ ^]^ Copyright 2015, Nature Publishing Group. B) Surface modification of the PDMS microchannel with collagen gel. Left image scale bar 100 µm, others 200 µm. Reproduced with permission.^[^ ^281a^ ^]^ Copyright 2016, PLOS. C) Circular, hollow endothelial monolayer formed with 3D collagen matrix using 3D printed tubes. Scale bar 200 µm. Reproduced with permission.^[^ ^292^ ^]^ Copyright 2015, AIP Publishing.

The 2.5D microfluidic models consist of a suitable hydrogel matrix for the cell support to create a hollow endothelium lumen within a microfluidic channel. The Young's modulus of the PDMS varies from 500 kPa to 4 MPa, which is extremely high in comparison to that experienced by the endothelial cells in the brain capillaries (i.e., 1 kPa). Hence, surface modification of PDMS channels usually involved coating of microchannels with poly‐l‐lysine or collagen to mimic the extracellular matrix (ECM) (**Figure** **16**A).^[^ ^276^ ^]^ Functionalization of the PDMS microchannel by covalently binding collagen to the surface to develop the suitable ECM for endothelial cells was also demonstrated (Figure 16B).^[^ ^281a^ ^]^ Endothelium monolayer models that form a hollow, circular lumen have been shown to be more appropriate BBB models than the flat 2D models to investigate neuroinflammatory diseases such as cerebral ischemia (caused by lack of oxygen and glucose), which is mediated by proinflammatory cytokines such as tumor necrosis factor (TNF‐*α*).^[^ ^276^ ^]^

2.5D microfluidic BBB models have been tested with polymeric nanoparticles. For example, commercially available human microfluidic BBB model (μHUB) was used to investigate the influence of nanoparticle size, shape, and their plastic elasticity on the ability for particles to cross the BBB.^[^ ^287a^ ^]^ The endothelial cells were seeded in such a way that they form a continuous, hollow lumen of the monolayer throughout the rectangular microchannel. Rod‐shaped stiff carboxylated polystyrene and soft PEG‐diacrylate nanoparticles were synthesized and tested in the membrane‐less parallel PDMS microfluidic design of μHUB model. This study demonstrated that transport of smaller, stiffer, spherical nanoparticles across the BBB was more efficient over larger, softer, and rod‐shaped nanoparticles. The nanoparticles interacted with the hollow lumen of the endothelial monolayer in two distinct yet coupled events—adhesion followed by basolateral transport based on their size and shape.

3D printing has also been used in 2.5D BBB models to replicate the curvature, physiological cyclic stretch, and porosity of the blood vessels. For example, a microfluidic BBB model was developed using 3D printed rods embedded in collagen type I matrix to mimic the curvature of blood vessels (Figure 16C).^[^ ^292^ ^]^ Murine endothelial cells (bEnd.3) formed a monolayer lumen on this collagen matrix. Similarly, using two‐photon laser lithography, a porous tubular structure was fabricated to recapitulate the capillaries of the neurovascular unit.^[^ ^293^ ^]^ This biohybrid, biomimetic BBB model consisted of a porous tubular structure (10 µm diameter, pore size 1 µm). However, low TEER value of 75 Ω·cm^2^ with mouse brain endothelial cells (bEnd.3) was reported similar to 2D Transwell models.^[^ ^293^ ^]^ While some of the 2.5D models offered only shear stress contribution, cyclic stretch or pulsatile radial strain generated due to pressure‐induced dilation was also identified as a mechanical stimulus experienced by the endothelial cells.^[^ ^255a^ ^]^ Pulsatile‐driven convective flow induces retrograde transport of molecules along the basement membrane in the direction of flow, resembling the transport of waste products in the brain.^[^ ^255a^ ^]^

#### 4.2.6 3D Microfluidic Models

**Figure 17:**
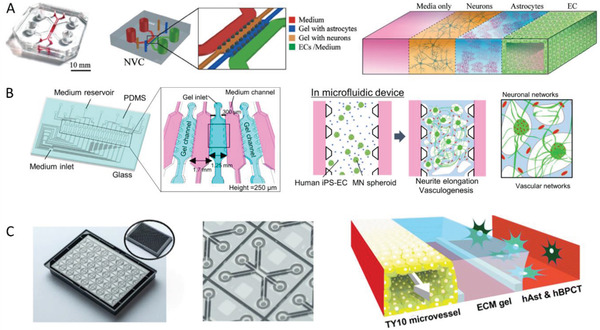
Examples of dynamic 3D microfluidic BBB models. A) BBB microfluidic device showing interconnected microchannels separated with pillar structures. Schematic showing the central two microchannels culturing neurons and astrocytes in a 3D hydrogel and the outer channel comprises of a hollow lumen of endothelial cells. Reproduced with permission.^[^ ^282^ ^]^ Copyright 2017, Royal Society of Chemistry. B) BBB microfluidic device showing alternate microchannels for gel and perfusion separated by pillars. Human iPSC‐endothelial cells are seeded along with motor neuron spheroids to form vascular and neuronal network. Reproduced with permission.^[^ ^277^ ^]^ Copyright 2018,Wiley‐VCH C) Organoplate, a three‐lane microfluidic chip fabricated within a 384‐well microtiter plate, and the channels containing gel and medium are separated by phaseguides fabricated within the microfluidic device. Reproduced with permission.^[^ ^295^ ^]^ Copyright 2016, Nature Publishing Group.

The 3D microfluidic model consists of a hollow lumen of endothelial monolayer interacting with the end‐feet of astrocytes and together with neuronal cells cultured in hydrogel to maintain their 3D morphology. Establishing a complete NVU model is crucial to investigate the pathological conditions of neurological disorders. 3D microfluidic BBB chips have been developed to include either primary or induced pluripotent stem cell (iPSC)‐derived endothelial cells. Co‐culture of endothelial cells with neurons in 3D configuration demonstrated increased expression of ZO‐1 (**Figure** **17**A).^[^ ^282^ ^]^ In addition, the endothelial cells also played a crucial role in enhancing the neuronal function and synaptic transmission, and neuron outgrowth. The presence of neuronal synapses, astrocytic network, and independent supply of culture media in 3D microfluidic BBB models have shown BBB‐specific features that mimic the in vivo physiology (Figure 17B).^[^ ^277^, ^282^, ^294^ ^]^ These kind of 3D models that mimic the angiogenesis and neurogenesis are useful for translational research and high‐throughput drug screening.

In another 3D microfluidic model,^[^ ^296^ ^]^ the middle, interconnected microfluidic channel was seeded with human iPSC‐ECs, co‐cultured with pericytes and astrocytes to form a self‐assembled 3D microvascular network of BBB within a fibrin hydrogel flanked by two channels cultured with endothelial cells. This model showed the interaction of astrocytic end‐feet with the 3D vascular network in the 3D matrix, which resulted in the upregulation of transporter proteins, such as GLUT‐1. Polystyrene and polyurethane nanoparticles conjugated with transferrin were perfused in this model to study the particle uptake, spatiotemporal distribution in the 3D microvasculature, and permeability of nanoparticles across the BBB.^[^ ^297^ ^]^ The iPSC‐ECs showed internalization of the nanoparticles through TfR‐mediated transcytosis with the nanoparticles localized near the cell nuclei. This suggested that nanoparticles were likely to be packaged in vesicles and trafficked out of the endothelial cells.

A commercially available microfluidic chip, Organoplate,^[^ ^298^ ^]^ has been employed to develop 3D models of BBB‐on‐chip,^[^ ^295^ ^]^ glioma‐on‐chip,^[^ ^299^ ^]^ gut‐on‐chip,^[^ ^300^ ^]^ and vessel‐on‐chip.^[^ ^301^ ^]^ Organoplate is a three‐lane microfluidic chip fabricated in microtiter plate format comprising 96 tissue chips that can be used for high‐throughput drug screening studies. This three‐lane microfluidic chip has small protrusions called phaseguides to separate channels instead of using porous membranes or pillar structures to interconnect two flanking parallel microchannels (Figure 17C).^[^ ^295^ ^]^ One of the three channels forms an endothelial monolayer against a 3D hydrogel (second channel). Astrocytes and pericytes added to the third channel complete the BBB‐on‐a‐chip model. Although Organoplate is a commercially available BBB model, it is limited to study particle transcytosis events due to the considerably large central lane (hundreds of microns) in comparison to the in vivo basement membrane thickness (tens of nanometers). The thick ECM hydrogel layer (second channel) limits the diffusion of nanoparticles and other material transport across the BBB from the endothelial channel. This limitation has been highlighted in a follow up study using mouse monoclonal antibody (MEM‐189) conjugated with anti‐human transferrin receptor perfused in an Organoplate BBB model.^[^ ^302^ ^]^ The murine antibody demonstrated active transport mediated by the transferrin receptor within 1 h.

## 5 Current Research Challenges and Future Perspectives

The nanomedicine landscape is evolving rapidly, and new nanoparticle formulations are continuously being investigated in pre‐clinical and in clinical trials. Some nanomedicine candidates have been successfully transitioned into the clinical practices,^[^ ^66^ ^]^ and polymeric nanoparticles have made a notable development with over ten formulations currently under clinical trial testing.^[^ ^66^ ^]^ In relation to the treatment of neurological disorders, no nanoparticle formulations have received approval thus far. However, a cationic liposome (SGT‐53) for gene therapy is being investigated for recurrent glioblastoma and CNS malignancies in clinical trials.^[^ ^66^ ^]^ Current nanoparticle formulations have yet to show success for CNS drug delivery in the clinic, and this is attributed to the complexities of drug delivery to the CNS and in particular to cross the BBB. A drug delivery system needs to be specifically designed to overcome the BBB and reach the brain tissues. Polymeric nanoparticles are especially well‐suited to carry out this task due to the unique control over particle properties including engineering particle size, grafting BBB targeting agents, and controlling drug release profiles.

Recently, a number of new promising BBB targeting moieties have been discovered, including plasma proteins, antibodies, peptides, aptamers, and small molecules. A direct comparison of their performance is difficult due to the presence of several variables, such as the surface density of the ligand, nanoparticle size, the testing model (in vitro or in vivo), and characterization methods chosen. Nevertheless, intermediate affinity antibodies and LDL receptor family targeting peptides, such as angiopep‐2 and ApoE, have shown better outcomes. For example, paclitaxel conjugated angiopep‐2 has been tested in phase II clinical trials for brain metastases,^[^ ^303^ ^]^ and iduronate‐2‐sulfatase conjugated to an anti‐transferrin‐receptor is currently under human clinical trials to treat Hunter syndrome.^[^ ^304^ ^]^ Nevertheless from a safety perspective, it is important to consider the acute and chronic effects of targeting brain receptors, for example, determining if nutrient transport and uptake is affected by targeting the transferrin or insulin receptor.^[^ ^150^ ^]^ Furthermore, penetrating the BBB is only half of the story as additional targeting to the diseased site often is required, for example, in glioblastoma. Therefore, to use multiple types of ligands (i.e., dual‐targeting strategy) or ligands that target receptors, which are highly expressed on both the BBB and targeted cells such as angiopep‐2 and transferrin for brain cancers could be the game changer. The size of nanoparticles plays a less important role in BBB transfer compared to surface functionality. However, particles that are smaller than 100 nm tend to penetrate deeper into the brain parenchyma. Nevertheless, the nanoparticle size is instrumental to evade renal clearance as well as limit uptake by the MPS, which indirectly influences the chance on BBB transcytosis. A less studied aspect is the nanoparticle shape, and more investigations are needed to fully harness the potential benefits.

Other methods to enhance the CNS delivery of polymeric nanoparticles also show promise in preclinical studies. For example, focused ultrasound is an emerging treatment method that leverages acoustic energy to oscillate administered microbubbles resulting in a temporarily disruption of the BBB.^[^ ^305^ ^]^ This disruption can be regionally targeted using MRI guidance. Moreover, focused cranial radiation therapy is able to modulate the tumor BBB and has been shown to improve the uptake of PEG‐*b*‐P(CL‐*co*‐LA) nanoparticles in glioblastoma,^[^ ^306^ ^]^ and convection‐enhanced delivery even bypasses the BBB and can enhance nanoparticle distribution by utilizing hydraulic pressure to deliver an infusate directly into a target region.^[^ ^11^ ^]^ Nevertheless, additional studies are needed to further explore these methods in a clinical setting.

Despite the species‐to‐species differences, in vivo models are still commonly used to test the efficacy of nanoparticle to cross the BBB. Advanced imaging techniques have been exploited to accurately trace the in vivo fate of polymeric nanoparticles. Although fluorescence labeling is often used due to the ease of conjugation protocols and the availability of different wavelengths, the fluorescence has a limited penetration depth, which prevents quantification and real‐time non‐invasive assessment in large animals and human. In contrast, radioisotopes have an unlimited penetration depth and their concentration can be quantitatively assessed in vivo via PET imaging or ex vivo using a gamma counter. Results should be assessed carefully, nevertheless, as nanoparticle delivery does not equate the delivery and release of the loaded pharmaceutical, and PD studies are essential, either by means of histology or in vivo imaging using PET or MRI. It is also very important to consider carefully which disease model is the most suitable. For example, although subcutaneous brain cancer models are easy to set up, they do not recapitulate a BBB and are therefore not clinically relevant. Moreover, the physiology of the BB(T)B in orthotopic brain tumors depends on the chosen brain cancer cell and tumor size. Where some cancer cell lines result in a very dysfunctional and “leaky” BB(T)B that enables any nanoparticle to pass, others have an intact BBB, which more closely resembles lower grade diffuse gliomas and the periphery of clinical glioblastoma.

In vitro models have significantly improved and transitioned from inadequate static BBB models to dynamic microfluidic models. These so called “organ‐on‐a‐chip” systems enable the investigation of the human‐derived cells rather than extrapolation from an animal model. With the advanced engineering of in vitro models, cellular microenvironment (e.g., 3D co‐culture) and mechanical stimuli (e.g., shear stress) can be accurately recapitulated. These in vitro BBB models are able to mimic the neurovascular unit, study the transport of nanoparticles across the BBB and perform high‐throughput analysis.^[^ ^259^ ^]^ There has been an intense development in relation to the synthetic and biological materials used for microfluidic chips. For example, conventional stiff porous membranes have been replaced with ultrathin silicon nitride and polycarbonate membranes with the aim to reproduce the in vivo mechanical cues.^[^ ^286^, ^307^ ^]^ About 36 immortalized endothelial cell lines have been investigated for in vitro models to mimic the in vivo cell–cell communication and cell–ECM interface of the BBB.^[^ ^250^ ^]^ Although, cell lines of rat, bovine, and porcine have been employed to develop BBB models, human immortalized cell lines are considered as the ideal cell line to mimic the human brain system. Currently, there is no standard range of TEER values due to wide variation in the setup of BBB models. It is crucial to further optimize the culture conditions for the cell lines to improve their application in BBB models. Stem cells have been shown to form a tight barrier in in vitro models with reported TEER values similar to physiological conditions and have the potential to be developed into personalized medicine scope by using patient‐derived stem cells to achieve patient‐specific BBB models for drug and nanoparticle testing. Human iPSCs can be cultured for an extended duration on 2D substrates. However, their exact behavior and phenotype remains to be fully characterized, and 3D culture of iPSCs in microfluidic models are preferred to immortalized cell lines, as they closely mimic the human microphysiological BBB system.^[^ ^26b^ ^]^

3D microfluidic models are particularly interesting and relevant for the study of BBB development and pathologic conditions. Some of the organ‐on‐a‐chip devices are now commercially available to develop in vitro 3D co‐cultures for drug screening studies. For example, Organoplate (Mimetas BV, Leiden) has developed a stratified array of 96 microfluidic chips with phase guiding technology and integrated these into a 384‐well titer plate to study the differentiation of neuroepithelial stem cells into functional dopaminergic neurons.^[^ ^308^ ^]^ In addition, SynVivo commercialized a 3D microvascular network named SynBBB that has been employed to develop an in vitro neo‐natal BBB model.^[^ ^26^, ^309^ ^]^ Hesperos Inc., (Orlando, USA) also commercialized a BBB microfluidic chip tailored for culturing human‐derived iPS cells to investigate the transport mechanisms through the BBB.^[^ ^310^ ^]^

A wide range of in vitro microfluidic BBB models have been developed and these have demonstrated their proof‐of‐concept value, but there is still a dire need for an integrated NVU model that fully mimics the in vivo physiology. The overarching goals for in vitro BBB models is to reduce and replace animal studies and to fast track the development of novel drug or drug/nanoparticle formulation testing and the translation into clinical practices. There is still a lot of work required for chip validations and regulatory adaptations before such BBB in vitro models can be fully integrated into the mainstream drug‐testing pipeline. Moreover, integration with in silico computation and machine learning algorithms^[^ ^311^ ^]^ could streamline the nanoparticle/drug complex development for BBB targeting, and eventually replace the in vivo studies by cheaper and more predictable methods.

## Conflict of Interest

The authors declare no conflict of interest.
